# Tuft dendrites of pyramidal neurons operate as feedback-modulated functional subunits

**DOI:** 10.1371/journal.pcbi.1006757

**Published:** 2019-03-06

**Authors:** Florian Eberhardt, Andreas V. M. Herz, Stefan Häusler

**Affiliations:** 1 Bernstein Center for Computational Neuroscience Munich, Germany; 2 Faculty of Biology, Ludwig-Maximilians-Universität München, Germany; University College London, UNITED KINGDOM

## Abstract

Dendrites of pyramidal cells exhibit complex morphologies and contain a variety of ionic conductances, which generate non-trivial integrative properties. Basal and proximal apical dendrites have been shown to function as independent computational subunits within a two-layer feedforward processing scheme. The outputs of the subunits are linearly summed and passed through a final non-linearity. It is an open question whether this mathematical abstraction can be applied to apical tuft dendrites as well. Using a detailed compartmental model of CA1 pyramidal neurons and a novel theoretical framework based on iso-response methods, we first show that somatic sub-threshold responses to brief synaptic inputs cannot be described by a two-layer feedforward model. Then, we relax the core assumption of subunit independence and introduce non-linear feedback from the output layer to the subunit inputs. We find that additive feedback alone explains the somatic responses to synaptic inputs to most of the branches in the apical tuft. Individual dendritic branches bidirectionally modulate the thresholds of their input-output curves without significantly changing the gains. In contrast to these findings for precisely timed inputs, we show that neuronal computations based on firing rates can be accurately described by purely feedforward two-layer models. Our findings support the view that dendrites of pyramidal neurons possess non-linear analog processing capabilities that critically depend on the location of synaptic inputs. The iso-response framework proposed in this computational study is highly efficient and could be directly applied to biological neurons.

## Author summary

Pyramidal neurons are the principal cell type in the cerebral cortex. Revealing how these cells operate is key to understanding the dynamics and computations of cortical circuits. However, it is still a matter of debate how pyramidal neurons transform their synaptic inputs into spike outputs. Recent studies have proposed that individual dendritic branches or subtrees may function as independent computational subunits. Although experimental work consolidated this abstraction for basal and proximal apical dendrites, a rigorous test for tuft dendrites is still missing. By carrying out a computational study we demonstrate that dendritic branches in the tuft do not form independent subunits, however, their integrative properties can be captured by a model that incorporates modulatory feedback between these subunits. This conclusion has been reached using a novel theoretical framework that can be directly integrated into multi-electrode or photo-stimulation paradigms to reveal the dendritic computations of biological neurons.

## Introduction

A key objective of theoretical neuroscience is the development of simplified neuron models that incorporate relevant features of neuronal function while abstracting away nonessential complexity. According to the classical point-neuron assumption, synaptic inputs to a neuron sum linearly at a single integrative node, the soma, where the resulting membrane potential is transformed non-linearly to generate the neuronal response [1, 2]. Although this simplified description might approximate the dynamics of certain neuron types, it is challenged for pyramidal cells that exhibit complex morphologies and spatially modulated distributions of ion channels (for recent reviews see [3, 4]). These characteristics generate regenerative events, such as Na^+^ or NMDA (N-methyl-*D*-aspartate) spikes, which are localized in specific branches or subtrees so that the neuron can no longer be described by a single voltage compartment.

It has been previously proposed that individual dendritic branches act as independent functional subunits within a two-layer feedforward model [5–7]. The first layer represents the computations of the individual branches, i.e., the voltage responses to local synaptic inputs, which are linearly summed and passed through a final non-linearity representing the second layer. Experimental and simulation studies consolidated the two-layer model as a suitable abstraction of synaptic integration in basal and proximal apical dendrites [8–11].

Häusser and Mel [12] suggested that even the distal apical tuft may function as a separate two-layer model that feeds into a proximal two-layer model, but a rigorous test of this hypothesis is still missing. In support of it, uncaging experiments have shown that NMDA spikes elicited in the fine branches of tuft dendrites generate voltage transients that are largely confined to the same branch and strongly attenuated across branches [13]. However, NMDA spikes in different branches can sum to initiate an apical calcium spike that propagates back into the distal branches. The impact of this electrical coupling on the core assumption of the two-layer model—subunit independence—remains unclear.

Related work [11] has shown that synaptic inputs to nearby branches in tuft dendrites cannot be described by a two-layer model if its integrative node is located at the soma. This observation hints at a partial breakdown of the functional independence of individual branches but might be attributed to an additional non-linearity between the site of the summation of the subunit outputs and the soma—the calcium spike initiation zone. Estimating this non-linearity poses a major problem for tests of the two-layer hypothesis. It requires that the result of the summation of the subunit outputs is known. But this information is rarely available because of the difficulty to record from the fine dendritic branches in the tuft.

This obstacle can be bypassed by iso-response methods. For this approach inputs to a system are varied such that a chosen output measure stays constant. As shown by various examples in different neural systems, iso-response methods can readily be integrated into neurophysiological experiments (see [14]). All that is needed is a closed-loop setup with which measurements of the neural output variable can be used to tune the inputs. The stimuli that result in constant outputs define “iso-response curves” that are independent of the specific form of the system’s final non-linearity. An estimation of this non-linearity is not required. Here, we show that the shape of the subunit non-linearities of a two-layer model can be read off from only a few iso-response curves, and that the validity of a two-layer model can be assessed by additional test iso-response curves.

We apply this theoretical framework to a detailed multi-compartment model of a CA1 pyramidal cell [6] and investigate the dendritic integration properties of different parts of the apical dendrites including proximal subtrees and the tuft. For this purpose, we analyze sub-threshold somatic membrane-potential responses to brief simultaneous synaptic inputs to pairs of dendritic branches. The two-layer model captures the somatic response to proximal inputs but fails for tuft dendrites. We generalize the two-layer model for the tuft, relax the core assumption of subunit independence, and incorporate additive and multiplicative feedback from a subsequent processing stage. This new model accurately predicts the somatic response to synaptic inputs on proximal apical branches as well as on tuft dendrites. The functional form and range of its non-linear feedback can be readily identified through the iso-response method.

## Results

**Fig 1:**
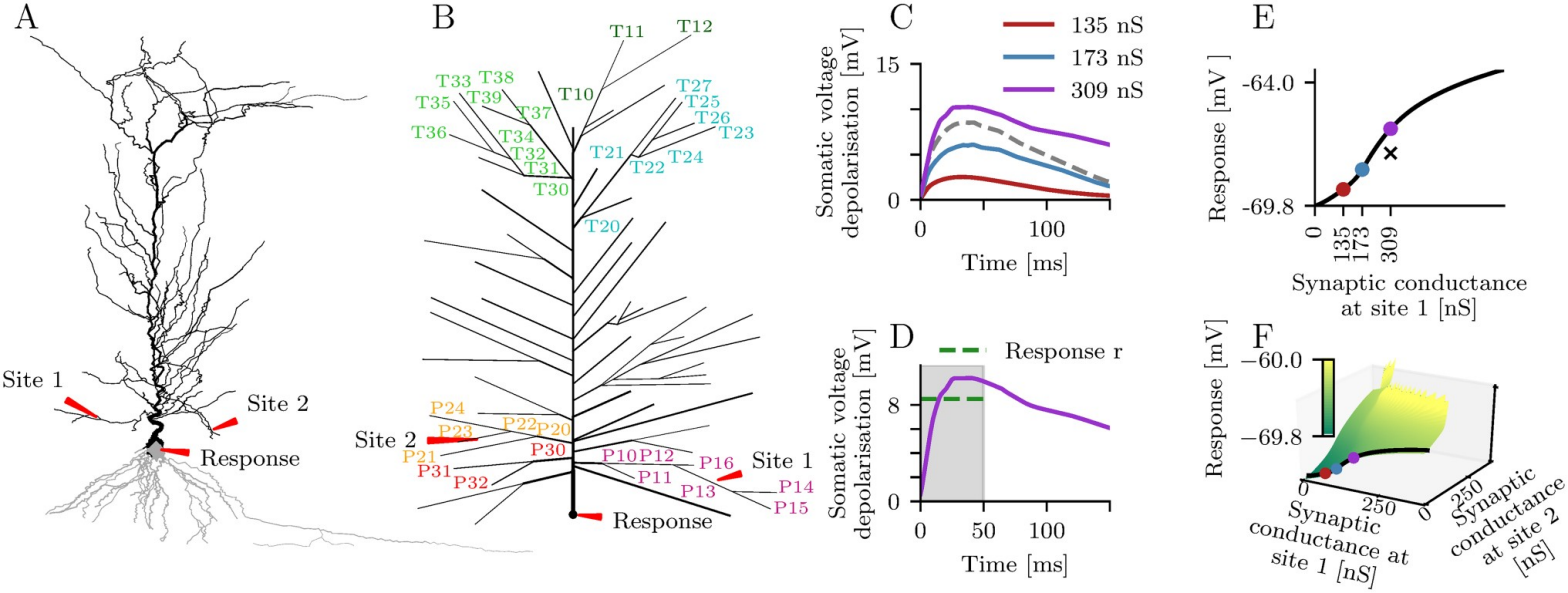
Detailed model of a CA1 pyramidal cell. (A) Morphology of the cell. The basal and the apical dendrite are shown in gray and black, respectively. Red arrows mark the location of the recording site and two dendritic branches receiving synaptic input. (B) Apical dendrite flattened in 2D. Branches are labeled according to their location in the proximal (P) dendrite or the tuft (T). The first number in a label denotes the subtree index and the second number denotes the branch index. (C) Somatic membrane depolarization for synaptic input at site 1 and three different peak conductance values. The depolarization for the highest peak conductance (purple line) is larger than the sum (dashed line) of the depolarizations for the two lower peak conductances (red and blue line) indicating supralinear dendritic integration. (D) The response *r* is defined as the membrane voltage averaged over 50 ms. (E) Dependence of the response on the synaptic peak conductance at site 1. The cross indicates the linear sum of the responses for the two lower peak conductances. (F) Dependence of the response on the synaptic peak conductances at site 1 and site 2.

We use a computational approach to study the non-linear dendritic integration of synaptic inputs. Brief input pulses are injected into pairs of dendritic branches of a detailed model of a CA1 pyramidal cell (Fig 1A and 1B). To investigate synaptic integration on a short time scale, we define the neuronal response *r* as the mean somatic sub-threshold membrane depolarization (Fig 1C) averaged over a time interval of 50 ms following synaptic stimulation (Fig 1D and 1E). The stimulation is repeated for different input strengths resulting in a two dimensional stimulus-response characteristic (Fig 1F). For each dendritic branch the maximum input strength is adjusted individually to obtain response values up to spike generation (the largest response values in Fig 1F indicate the spike threshold).

**Fig 2:**
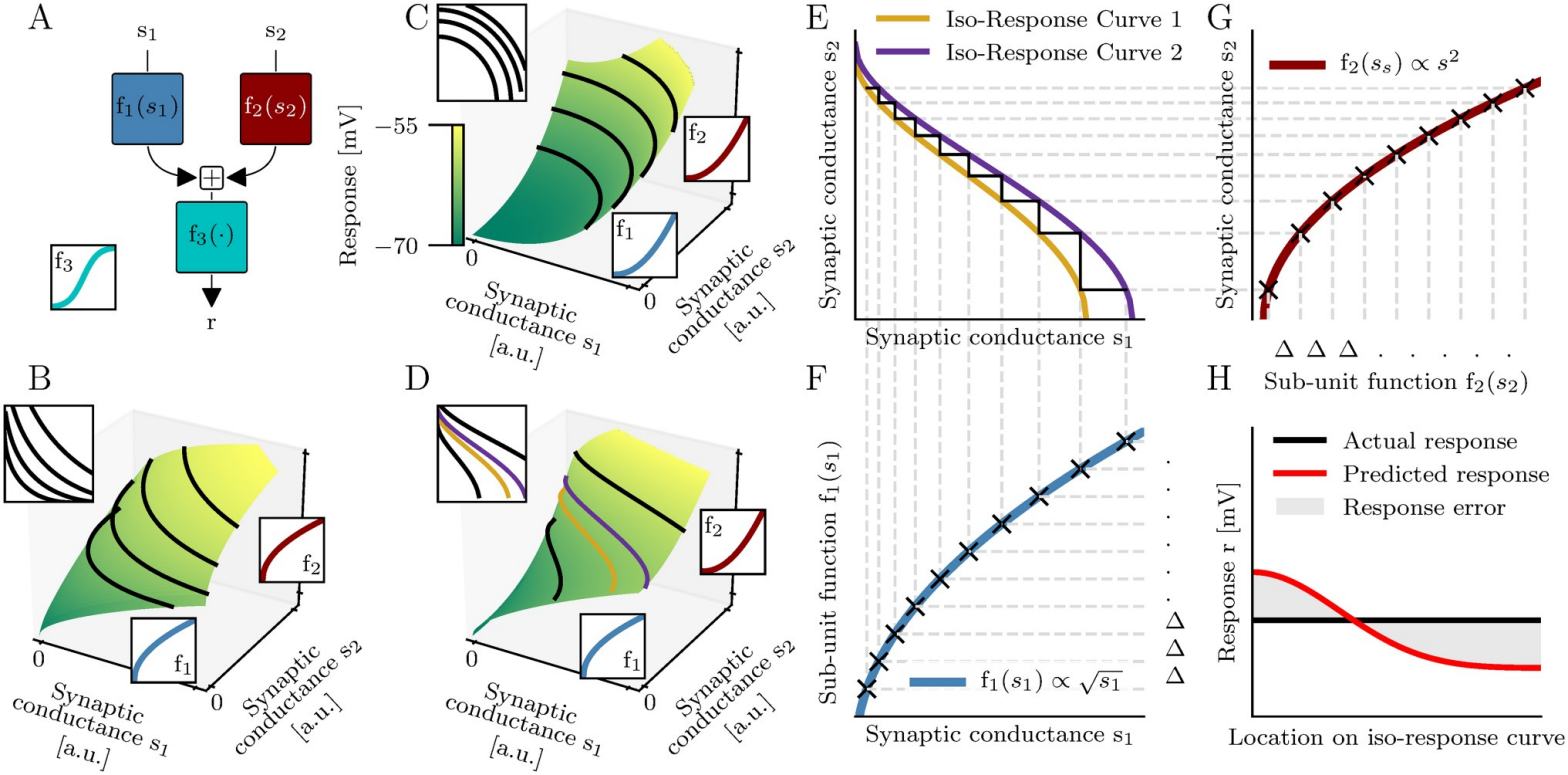
Identification of the feedforward two-layer model with iso-response methods. (A) Feedforward two-layer model. (B-D) Responses of three models consisting of different supralinear subunit non-linearities (bottom and right insets). Black, purple and yellow lines indicate iso-response curves. Top insets show iso-response curves from above. (E-G) Reconstruction of the first layer subunit non-linearities from the purple and yellow iso-response curves shown in D. (F,G) Black crosses mark reconstructed points on the graph of the subunit non-linearities. The blue and the dark red line show the true subunit non-linearities used to generate the response in D. Note that the axes in G are flipped and Δ denotes a constant step size. (H) The prediction error is measured by the variance of the predicted response on an additional test iso-response curve (not shown) normalized by the variance of the responses used for training (purple and yellow lines).

The feedforward two-layer model [7] describes the neuronal response by the following function

$$\begin{array}{c} r=f_{3}\left(f_{1}\left(s_{1}\right)+f_{2}\left(s_{2}\right)\right), \end{array}$$

where *s*_1_ and *s*_2_ denote the strengths of the two synaptic inputs (Fig 2A). The outputs of the individual subunits are modeled by the functions *f*_1_ and *f*_2_. A final non-linearity, denoted as *f*_3_, is applied to their sum.

For iso-response methods (see [14]) the input strengths *s*_1_ and *s*_2_ are varied such that the response *r* stays constant (Fig 2B). These stimuli define one dimensional “iso-response curves” in the two dimensional input space. Applied to a two-layer model of dendritic integration, the shapes of iso-response curves thus only depend on the subunit non-linearities *f*_1_ and *f*_2_ and not on the final non-linearity *f*_3_ (Fig 2B–2D), whereas the actual response *r* does depend on *f*_3_.

The subunit non-linearities of a two-layer model can be derived from only two iso-response curves (Fig 2E–2G and Materials and methods). A third iso-response curve can be used to test whether the response can indeed be described by a two-layer model. More precisely, the predicted test iso-response curve and the observed test iso-response curve are identical if and only if the observed responses can be described by a two-layer model. We assess the error of the two-layer model by the variance of the predicted responses on the observed test iso-response curve normalized by the response variance of the two training iso-response curves (Fig 2H). The test iso-response curve is located right below the spike threshold so that the two-layer model is investigated for its accuracy to generate single spikes. We successfully tested the iso-response method on artificial data (S1–S4 Figs) as well as for a pair of proximal dendritic branches with specified subunit non-linearities (S5 Fig).

### The two-layer model is accurate for proximal apical dendrites

**Fig 3:**
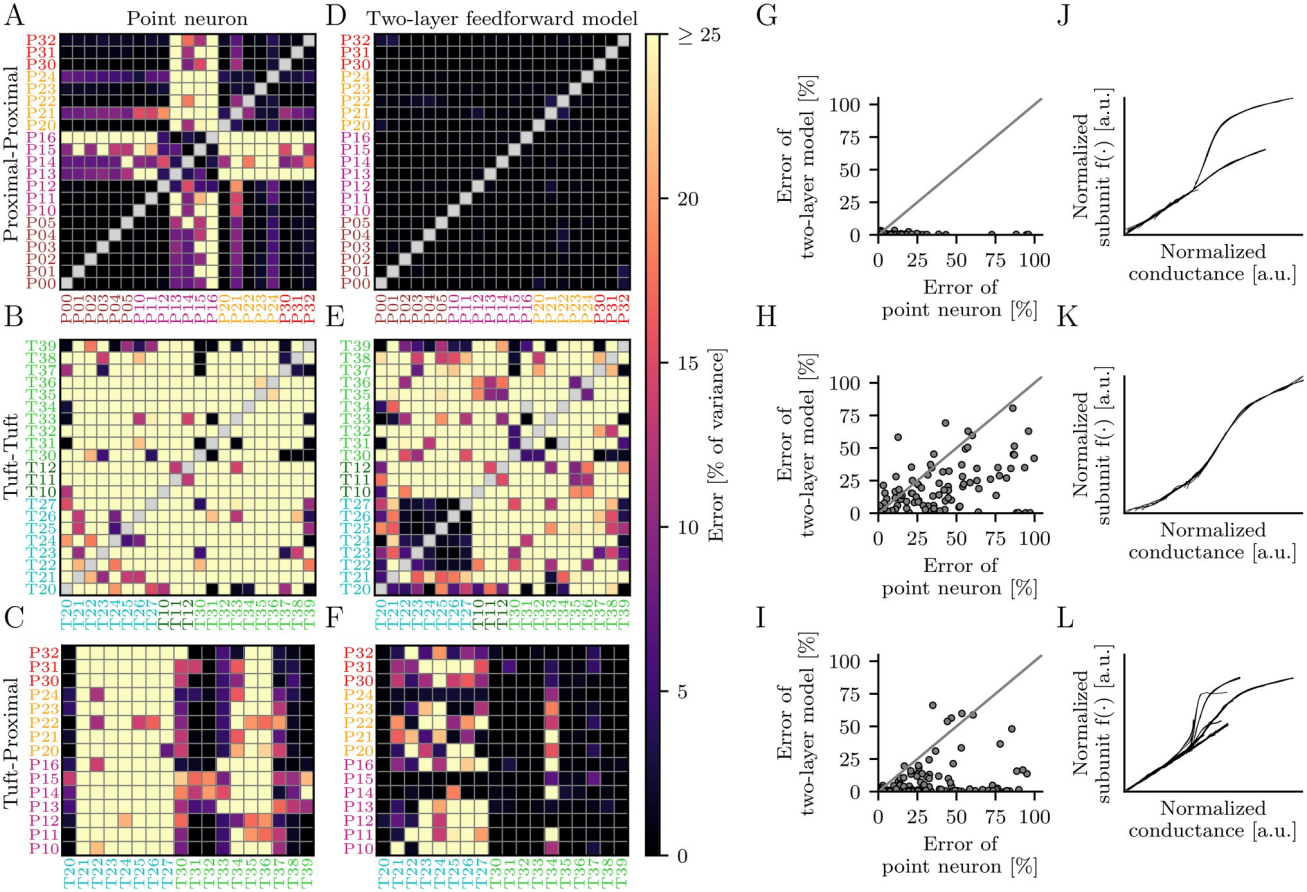
Performance of point-neuron models and feedforward two-layer models. (A-C) Prediction error of point-neuron models for pairs of P-P (A), T-T (B) and T-P (C) branches. The first number in a label denotes the subtree index and the second number denotes the branch index. Labels P00-P05 denote the segments of the proximal apical trunk, which are closest to the soma. (D-E) Prediction error of feedforward two-layer models. (G-H) Comparison of both models. (J-L) Normalized subunit non-linearities for two-layer models with low prediction error (< 4%) aligned to the onset of their non-linearity. The normalization involves a vertical and horizontal scaling and shift of each curve.

We stimulated pairs of branches located in the proximal (P) apical dendrites. First, we investigated for which branch pairs the somatic response can be successfully explained by a model that satisfies the classical point-neuron assumption. Here, synaptic inputs are summed linearly at the soma and subsequently transformed non-linearly to generate the neuronal response. We refer to this model as point-neuron model. We found that a large fraction of the proximal apical branches support linear dendritic integration (Fig 3A). In particular, all branches except P13-P16, P21 and P24 can be collected into one linear functional subunit.

However, non-linear dendritic integration occurs in the distal branches of two proximal apical subtrees. More precisely, the point-neuron hypothesis fails only if one of the stimulated branches is part of these subtrees and the other branch is not. Therefore, local synaptic input is first summed linearly within these subtrees and then transformed non-linearly to generate the somatic response.

Next, we tested the two-layer model for P-P branch combinations. We find that the model provides an accurate description of dendritic integration for all tested branch pairs with errors below 4% (Fig 3D). The reconstructed subunit characteristics are either linear or supralinear functions (Fig 3J) and can be divided into three classes (S6D and S13 Figs) according to their input impedance (S7 Fig). First, there are branches close to the trunk (S6A Fig) with approximately linear subunit functions. Second, there are branches with weak supralinearities where synaptic inputs trigger somatic spikes before the local depolarizations reach the threshold for dendritic non-linearities. Finally, there are branches with large local depolarizations (S7A Fig) that strongly attenuate from the dendrite to the soma (S7B Fig). These branches have supralinear subunit functions with only small linear onsets and rapid saturations as observed in previous simulation studies for distal synaptic input [11, 15]. The variability of the slopes of these functions reflects the differences in the distances from the stimulation sites to the soma [11]. Supralinear non-linearities occur only for those branches (P13-P16) for which the point-neuron model fails.

In an analogous manner, we tested the feedforward two-layer model for oblique branch combinations. We find that the feedforward model accurately predicts the somatic responses to synaptic inputs for all tested branch pairs (S12 Fig). Overall, the feedforward two-layer model is an accurate description of synaptic integration in proximal apical and oblique dendrites.

### The two-layer model fails for tuft dendrites

Next, we stimulated pairs of dendritic branches located in the proximal (P) apical dendrites and tuft (T) dendrites. The point-neuron model fails for nearly all T-T branch pairs (Fig 3B). The few exceptions might be attributed to the specific choices of test iso-response curves and presumably disappear for a different selection of curves.

Surprisingly, the majority of the T-T branch pairs is not well described by the two-layer model either, with errors mostly larger than 25% (Fig 3E). An accurate description is only possible within the distal dendrites of one particular subtree (T22-T27). However, if one branch is located outside of the subtree the model fails. The shapes of the normalized subunit non-linearities for the subtree T22-T27 are shown in Fig 3K (S14 Fig).

The point-neuron model fails for most T-P branch combinations as well. Only a few branch pairs consisting of a tuft branch close to the trunk and a linear proximal apical dendrite (as identified above) can be modeled by a point neuron. On the other hand, the two-layer model fails for one of the tuft subtrees (labels starting with T2) but mostly predicts the iso-response curves for the other subtree (labels starting with T3), apart from branch T34. The reconstructed subunit functions for branch pairs with low errors (<4%) resemble those observed for P-P and T-T branch pairs but incorporate additional non-linearities between the outputs of the dendritic branches and the site of their summation (Fig 3L and S15 Fig). Overall, the two-layer model fails to describe dendritic integration in the tuft.

### Feedback captures dendritic integration in the tuft

**Fig 4:**
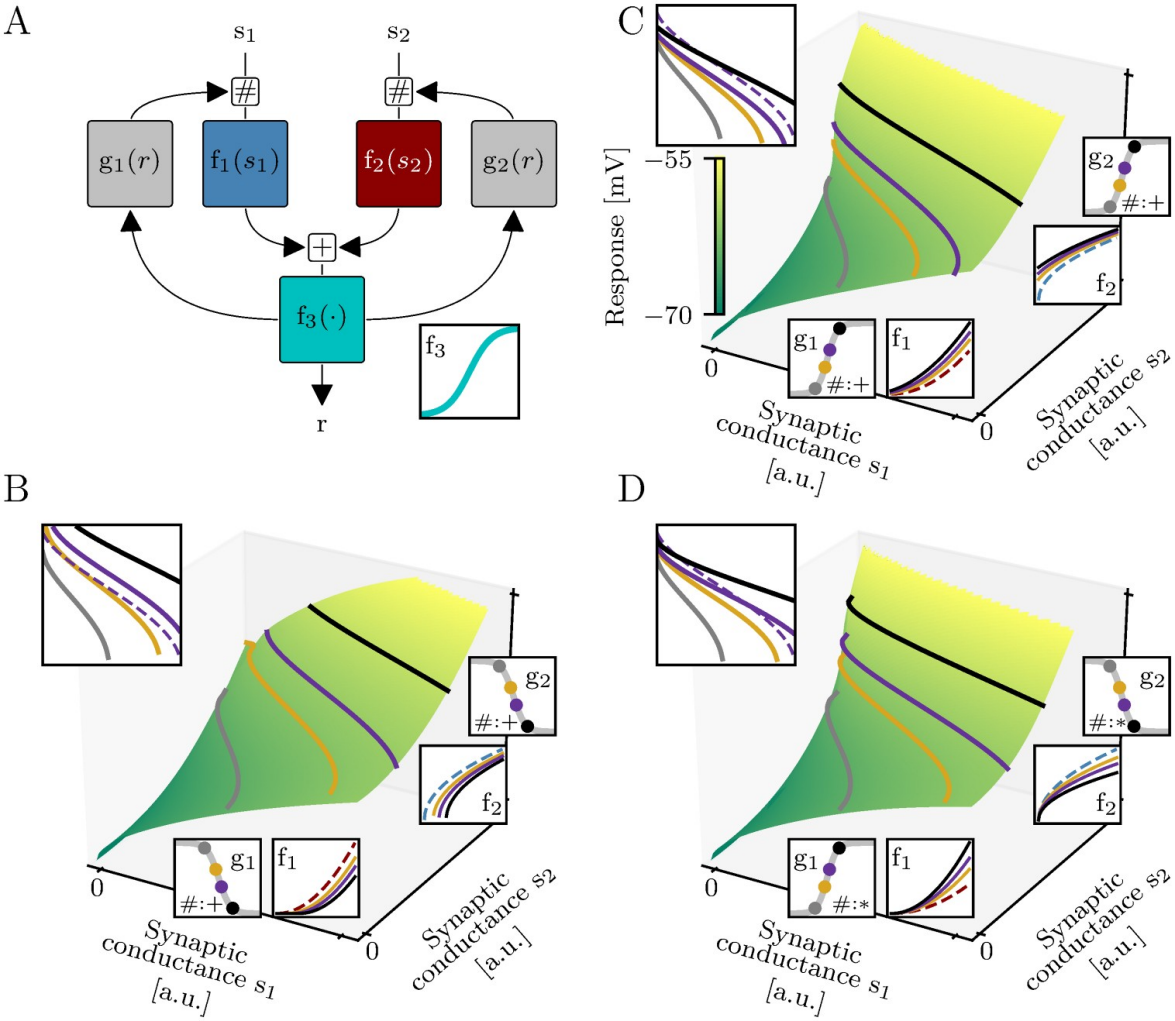
Two-layer model with feedback. (A) Feedback modulates the subunit inputs by an additive or multiplicative term (indicated by the placeholder #). These feedback terms are non-linear functions (*g*_1_ and *g*_2_) of the response. (B-D) Impact of feedback on the response for the two-layer model shown in Fig 3D. (B) Negative feedback shifts the iso-response curves further apart. (C) Positive feedback shifts the iso-response curves closer together. (D) Multiplicative feedback rescales the iso-response curves. Top insets show iso-response curves from above. Dashed lines illustrate the purple iso-response curve of the two-layer model without feedback (Fig 3D). Bottom and right insets show subunit non-linearities and feedback functions. The subunit non-linearities of the two-layer models without feedback are illustrated by the blue and the dark red dashed lines. Solid lines indicate the subunit non-linearities of the corresponding two-layer models with feedback for stimuli on three iso-response curves (black, purple and yellow lines). The feedback is constant on iso-response curves and shifts and rescales the subunit non-linearities. On the gray iso-response curves the feedback is zero.

We relax the assumption of subunit independence and generalize the purely feedforward model by feedback loops (Fig 4A). The somatic response is now given by the implicit function

$$\begin{array}{c} r=f_{3}(f_{1}(g_{1}^{\text{mult}}\left(r\right)\cdot s_{1}+g_{1}^{\text{add}}\left(r\right))+f_{2}(g_{2}^{\text{mult}}\left(r\right)\cdot s_{1}+g_{1}^{\text{add}}\left(r\right))), \end{array}$$

where $g_{i}^{\text{add}}$ and $g_{i}^{\text{mult}}$ denote additive and multiplicative feedback, respectively.

As the feedback is determined by the output *r* (see Fig 4A), it is constant along iso-response curves. Additive feedback shifts the iso-response curves in the stimulus space but does not change their shape. Negative feedback shifts iso-response curves further apart (Fig 4B), whereas positive feedback brings them closer to each other (Fig 4C). Multiplicative feedback rescales the iso-response curves in each dimension of the stimulus space (Fig 4D).

For two-layer models with feedback, we quantify the error as the variance of the predicted sum of the subunit non-linearities on the test iso-response curve. As before, we normalize by the corresponding variance of the two training iso-response curves. In contrast to the feedforward model, we now analyze the error of the argument of the final non-linearity *f*_3_ and not its output. The final non-linearity can not be estimated from *r* and the reconstructed subunit non-linearities, because the feedback is unknown for stimuli outside of the training and test iso-response curves. However, both error measures, i.e., the variance of the sum of the subunit non-linearities on the test iso-response curve and the response variance on the test iso-response curve, are qualitatively similar for the feedforward two-layer model (S8 Fig).

**Fig 5:**
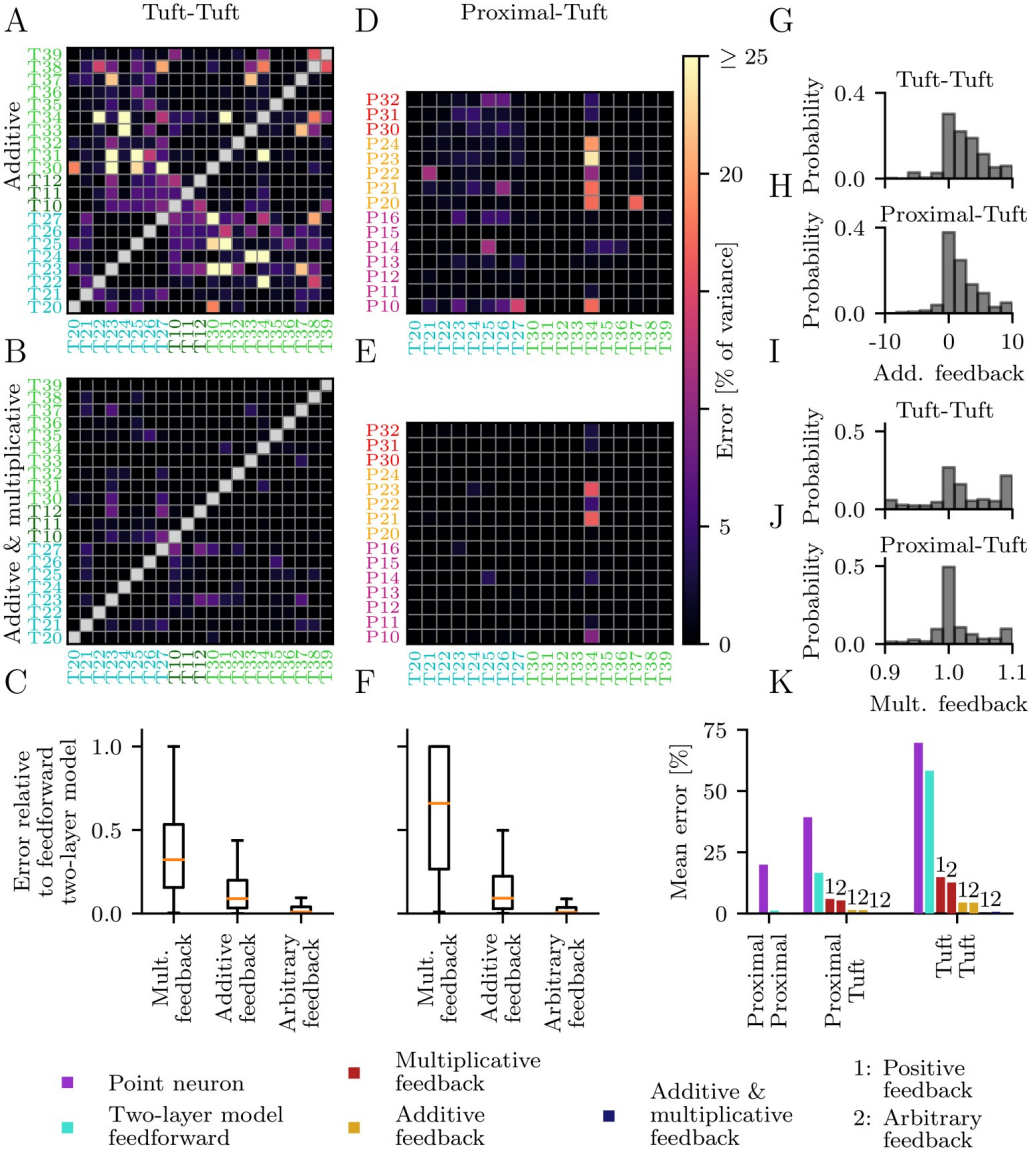
Performance of two-layer models with feedback. (A,D) Prediction error of two-layer models with additive feedback for T-T (A) and T-P (D) branch pairs. (B,E) Prediction error of two-layer models with additive and multiplicative feedback. (C,F) Errors of the two-layer models with feedback in units of the errors of the feedforward two-layer models. (G,H) Strength of additive feedback for T-T and T-P branch pairs. (I,J) Strength of multiplicative feedback for T-T and T-P branch pairs. (K) Performance comparison across models.

The extended two-layer model was applied to T-T and T-P branch pairs (Fig 5). Additive feedback is sufficient to explain synaptic integration for more than 72% of all branch pairs (4% threshold). In particular, it describes synaptic integration for all branch pairs within the same subtree (Fig 5A). Additional multiplicative feedback is required to achieve low prediction errors for a few pairs of branches from different subtrees (Fig 5B).

For T-P branch pairs additive feedback is sufficient to explain synaptic integration of more than 91% of all branch pairs (4% threshold). A combination of multiplicative and additive feedback further reduced the prediction error (Fig 5E).

In general, purely additive feedback results in lower prediction errors compared to purely multiplicative feedback (Fig 5C and 5F). The additive feedback is almost always positive and up to 10% of the input range (Fig 5G and 5H). This value has to be compared to the average distance between training iso-response curves of about 14% of the stimulus range. Restricting the additive feedback to only positive values has only an insignificant effect on the model error (Fig 5K). Furthermore, the feedback strengths are similar for T-T and T-P branch pairs.

The reconstructed multiplicative feedback values peak around 1 and are small (Fig 5I and 5J). A second peak around 1.1 for T-T branch pairs indicates that for some pairs stronger multiplicative feedback would further reduce the prediction error. However, we limited the multiplicative feedback values to a range such that the test iso-response curves were long enough to allow a reliable estimation of the model validity. Even within this limited range accurate two-layer models were identified. For a significant fraction of branches the multiplicative feedback values are below one.

A summary of the errors of all models is show in Fig 5K. A two-layer model without feedback accurately describes synaptic integration in proximal apical dendrites, but fails for the tuft. Additive feedback alone is sufficient to achieve a low mean prediction error. Restricting the additive feedback to positive values has only a minor effect on the performance.

**Fig 6:**
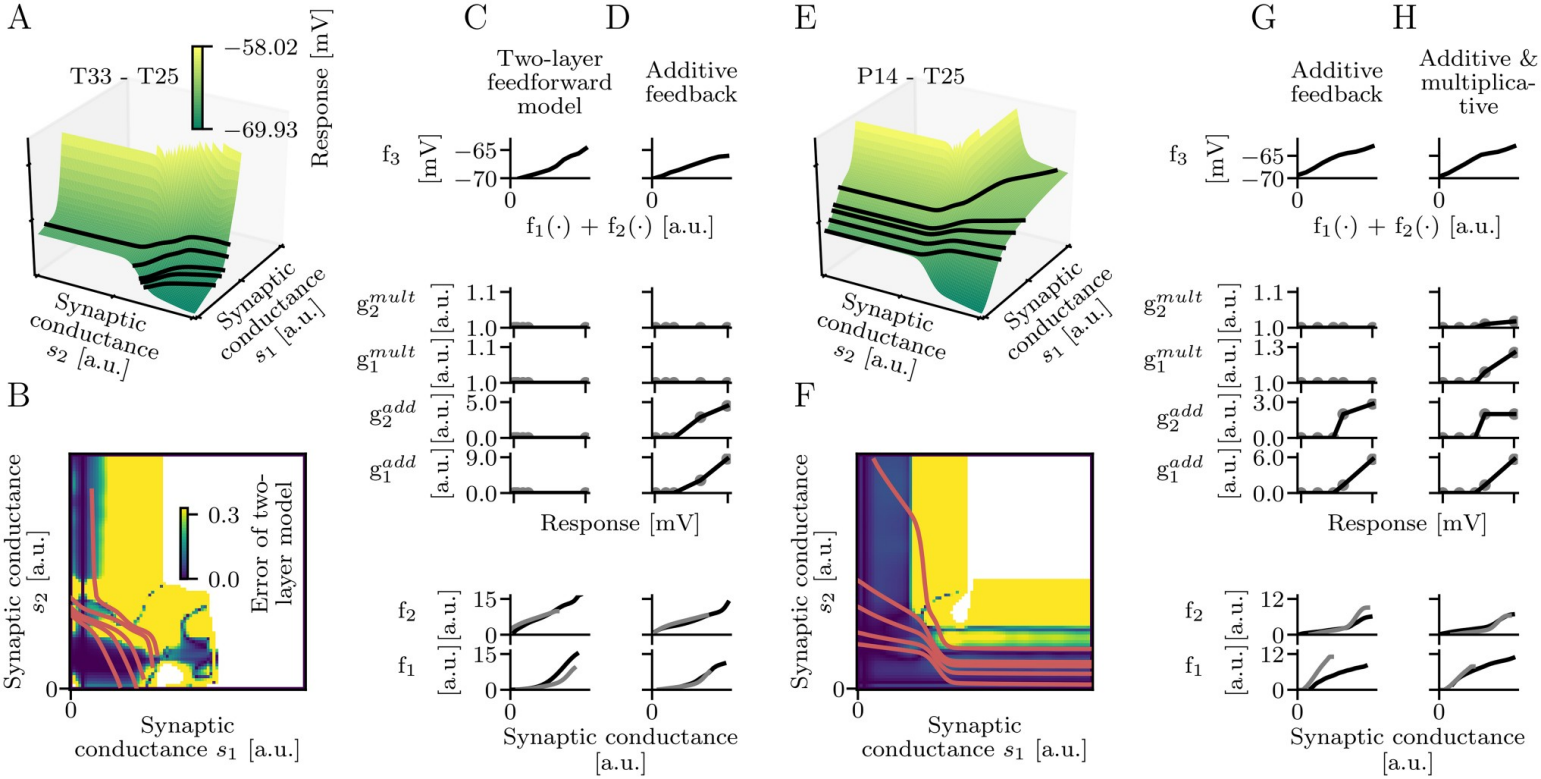
Examples of performance improvements through feedback. (A) Response for the branch pair T25-T33 and five iso-response curves (black lines). (B) Error of the feedforward two-layer model in dependence on the input regime. Yellow regions indicate failures of the feedforward two-layer model. The error is given by |log_2_(tan(*α*_*pred*_)) − log_2_(tan(*α*_*act*_))|. (C) Two reconstructions of each subunit non-linearity assuming a feedforward two-layer model. The two reconstructions of *f*_1_ differ by a horizontal shift. (D) Two reconstructions of each subunit non-linearity assuming a two-layer model with additive feedback. The reconstructed subunit non-linearities are nearly identical. The additive feedback compensates for the shift of *f*_1_ in C. (E) Response for the branch pair P14-T25 and five iso-response curves (black lines). (F) Error of the feedforward two-layer model in dependence on the input regime. (G) Two reconstructions of each subunit non-linearity assuming a two-layer model with additive feedback. Each of the reconstructed subunit non-linearities differs by a vertical rescaling. (H) Two reconstructions of each subunit non-linearity assuming a two-layer model with additive and multiplicative feedback. Again, the reconstructed subunit non-linearities are nearly identical. The multiplicative feedback compensates for the rescaling of *f*_1_ and *f*_2_ in G.

Fig 6 shows cases of branch pairs where dendritic integration can be described by a two-layer model with additive feedback but not without feedback (Fig 6A–6D), and where dendritic integration can be described by a two-layer model with additive and multiplicative feedback but not without multiplicative feedback (Fig 6E–6H). Fig 6B and 6F illustrate the input regime where the feedforward two-layer model without feedback fails. For both two-layer models two sets consisting each of three iso-response curves were used for training so that the reconstructed subunit non-linearities can be compared. For the branch pair where additive feedback is required the reconstructed subunit non-linearities of a two-layer model without feedback are shifted (Fig 6C). For the branch pair where multiplicative feedback is required the reconstructed subunit non-linearities of a two-layer model that implements only additive feedback are rescaled (Fig 6G). We have chosen feedback functions that compensate for both of these transformations. Moreover, the impact of this feedback on the response can be visualized as a shift and a rescaling of iso-response curves.

### Calcium currents disrupt the independence of dendritic subunits on short time scales

**Fig 7:**
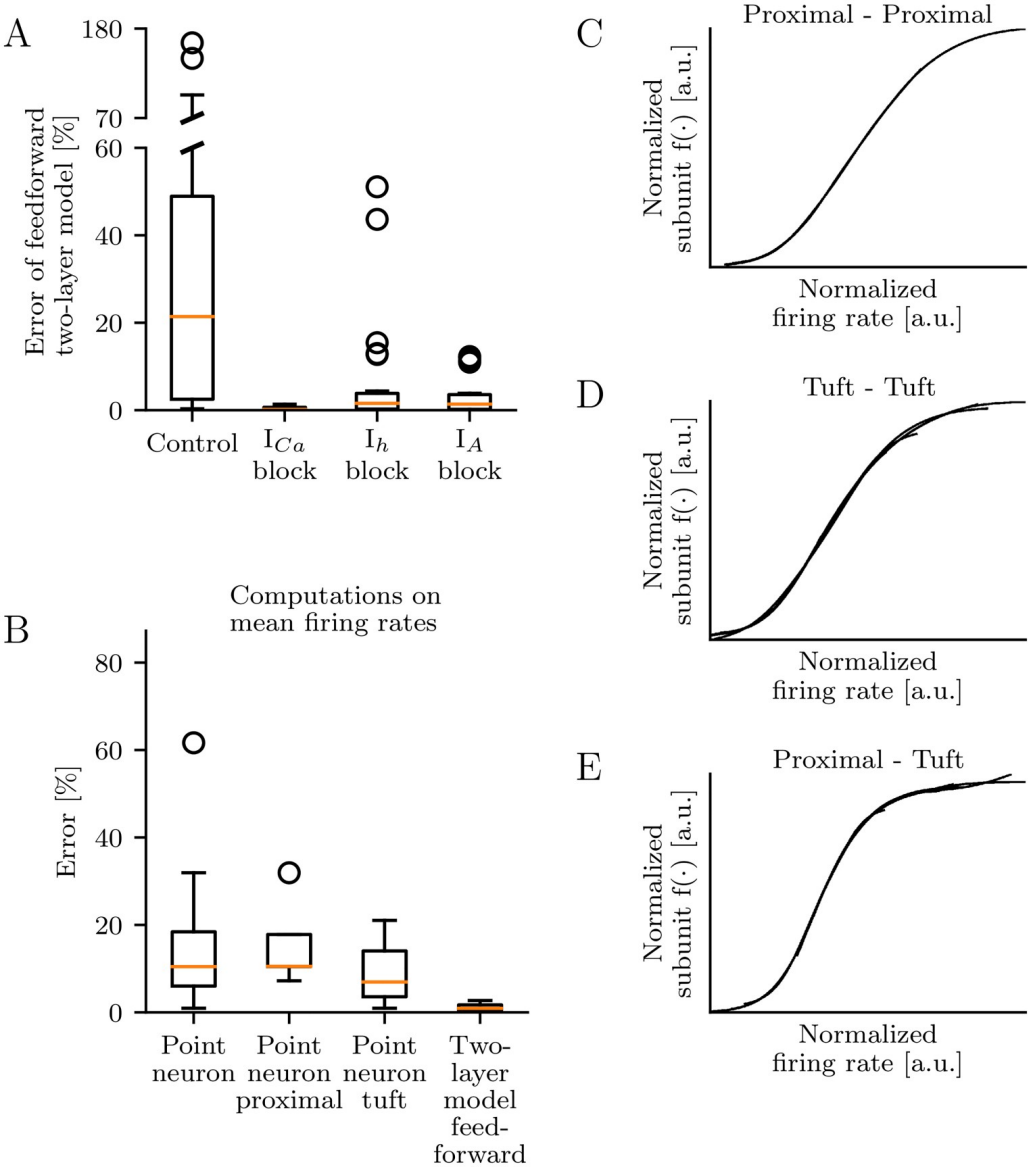
Calcium currents disrupt subunit independence. (A) Performance of feedforward two-layer models for a representative subset of branch pairs (see S9 Fig). Voltage-gated calcium currents (*I*_*Ca*_), an A-type potassium current (*I*_*A*_), and a hyperpolarization-activated cation current (*I*_*h*_) are blocked individually in the tuft. If voltage-gated calcium currents are blocked, dendritic integration can be explained by a feedforward two-layer model. (B) Performance of point-neuron and two-layer models that predict the somatic firing rate in response to synaptic Poisson input for the same representative subset of branch pairs as used in A. The feedforward two-layer model is sufficient to describe neuronal firing-rate responses even with intact *I*_*Ca*_. (C-E) Normalized subunit non-linearities for the feedforward two-layer models in B aligned to the onset of their non-linearity. The normalization involves a vertical and horizontal scaling and shift of each curve.

What is the biophysical basis for the feedback in the functional subunits of tuft dendrites? To answer this question, we repeat the simulation study for a representative subset of branches throughout the dendritic tree (S9 Fig) while blocking active ion channels exclusively present in the tuft. These are three voltage-gated calcium currents, an A-type potassium current and a hyperpolarization-activated cation current (*I*_*h*_). The block of each current improves the performance of the feedforward two-layer model but only the block of voltage-gated calcium currents results in close-to-optimal performance (Fig 7A). This finding indicates that calcium currents disrupt the independence of functional subunits in the tuft by shifting their non-linearities.

### Independent dendritic subunits for neuronal computations based on firing rates

In this study, we focus on dendritic integration on short time scales that are relevant for spike-timing codes. To test the generality of our findings for other neural coding scenarios we also investigate computations on spike counts within longer time intervals. Here, the input and the response of the pyramidal cell are encoded by the rate of synaptic input and the firing rate of the neuron, respectively. Ten synapses are located with equal spacing on each of two dendritic branches (for a representative subset of branches). All synapses on one branch are stimulated with homogeneous Poisson spike trains with identical rates and a duration of 500 ms. The input rates for the two branches varied individually up to a frequency of 40 Hz. The neuronal response, i.e. the firing rate of the pyramidal cell during stimulation, is averaged over 20 trials. As for computations on short time scales the point neuron model fails to predict the neuronal response to synaptic input to proximal as well as to tuft dendrites (Fig 7B). However, the feedforward two-layer model turns out to be sufficient to describe the firing-rate based non-linear integration. The reconstructed subunit functions for P-P and T-T branch pairs resemble those observed for precisely timed inputs (Fig 7C and 7D). In contrast, the subunit functions for P-T branch pairs (Fig 7E) do not incorporate additional non-linearities as found for precisely timed inputs (Fig 3L). Similarly, the iso-response method can be applied to other neural coding scenarios as long as the response variable is a continuous function of the inputs.

## Discussion

Using a detailed multi-compartment model of a pyramidal neuron and a novel theoretical framework based on iso-response methods, we investigated whether the somatic responses to simultaneous synaptic inputs to pairs of dendritic branches can be described by a two-layer feedforward model. We found that this two-layer model is an accurate description of dendritic integration in the proximal apical subtree. In contrast, the individual branches in the tuft do not implement independent functional subunits and synaptic integration cannot be described by a feedforward two-layer model. We extended the model and incorporated feedback from its integrative node back to the subunit inputs. We found that additive feedback interactions accurately predict the somatic response in many cases, sometimes multiplicative feedback is required.

### Underlying mechanism

Additive and multiplicative feedback terms correspond to horizontal shifts and horizontal rescalings of the subunit non-linearities, respectively. Various biophysical mechanisms have been associated with these operations (for a review see [16]). Shifts of dendritic non-linearities have been observed for additional current or conductance inputs in the proximity of a driving input [15, 17, 18]. In particular, it was shown for basal dendrites that distal modulatory input within the same branch mainly shifts the subunit non-linearities [15, 18]. In contrast, proximal modulatory input shifts and changes the slope of the subunit non-linearities. These asymmetric interactions have been linked to the voltage-dependence of NMDA channels and an increase in input resistance with increasing distance between the input and the soma.

Our results indicate that a similar modulation occurs across dendritic branches in the tuft. Horizontal shifts of the subunit non-linearities alone are sufficient to explain the response properties of most of the dendritic branches. However, for a small fraction of branches a change in the slope of their subunit non-linearities is required as well to describe the synaptic integration properties. This modulation may be either attributed to a change in the gain of the subunit non-linearity, which changes its maximum value as well, or a horizontal rescaling, which does not change the maximum value. Horizontal rescaling has been observed for modulatory input co-localized with driving input [18], whereas gain control was reported for proximal modulatory input [15, 18]. Here, we cannot discriminate between both cases for all branch pairs that implement multiplicative feedback. The subunit non-linearities were not driven to saturation for somatic responses below the action potential threshold, and no conclusion can be drawn about the change of their maximum value.

A major difference between the distal tuft and basal dendrites is the presence of hyperpolarization-activated cation currents (*I*_*h*_) and voltage-gated calcium channels. In agreement with experiments [13] we find that *I*_*h*_ currents facilitate subunit independence in the dendritic tuft. Moreover, our simulation study predicts that feedback interactions in tuft dendrites are mediated by calcium currents at the calcium spike initiation zone.

### Relation to previous work

Previous work [11] applied the two-layer model to predict the firing rate of a detailed pyramidal cell model in response to homogeneous Poisson input. This feedforward two-layer model correctly predicted the somatic response to a distant pair of apical tuft dendrites but failed for synaptic inputs to nearby branches in the tuft (see Fig S4 in [11]). In contrast, our results show that computations based on firing rates can be well described by a feedforward two-layer model even for tuft dendrites.

The failure of the two-layer feed-forward model in [11] to predict the firing-rate responses to synaptic inputs to nearby branches in the tuft can be attributed to either a disruption of the functional subunit independence or a distorted reconstruction of the model parameters. In [11], the final non-linearity of the two-layer model is approximated by the somatic f-I curve and the individual subunit outputs are assumed to contribute linearly to the total somatic current. If, under these conditions, the dendritic branches operate as independent functional subunits, their non-linearities can be estimated by a least-squares linear regression of the total somatic current on the synaptic inputs. However, for synaptic inputs to nearby distal branches, as shown in Fig S4C of [11], additional dendritic non-linearities occur between the location of the subunit output summation and the soma. In this case, the somatic current is no more a linear sum of the individual branch outputs and linear regression fails to correctly identify the dendritic non-linearities. As we find no evidence for a violation of the functional independence of dendritic branches in the tuft for computations based on firing rates our results suggest that the reduced performance of the two-layer model in [11] can be attributed to a distorted reconstruction of the final non-linearity.

The iso-response method has two advantages when compared to the method proposed in [11]. First, it does not require an estimate of the final non-linearity of the two-layer model that is rarely available for synaptic integration in the tuft because of the difficulty to record from fine dendritic branches. Once the subunit non-linearities of a feedforward two-layer model are identified its final non-linearity can be reconstructed from measurements of the somatic responses to single branch stimulations of varying strength. Second, iso-response curves are optimally efficient in that they are based on the smallest amount of data needed to identify the subunit non-linearities.

We have demonstrated that feedback in tuft dendrites impacts synaptic integration on the short time scales relevant for triggering single somatic spikes. The precise timing of spikes relative to hippocampal theta oscillations has been shown to carry information about the location of a rat within its environment [19, 20]. Moreover, recent work [21] supports the hypothesis that degraded spike timing in the hippocampus results in memory impairments.

In contrast, we found no evidence for the functional relevance of feedback for mean somatic responses, i.e. spike counts within longer time intervals. This difference might be attributed to two reasons. First, the brief synaptic input to pairs of dendritic branches was injected simultaneously, whereas the Poisson spike trains used for rate inputs were not synchronized. Simultaneous synaptic stimulation has been shown to enhance the non-linear integrative properties of dendrites [22]. Second, for brief synaptic stimulation we investigated somatic sub-threshold responses in contrast to multiple spikes for rate responses. As proposed in [11], action potential generation might facilitate subunit independence. Overall, our findings consolidate the feedforward two-layer model as a suitable abstraction of neuronal computations based on firing rates [5–12] but not on precisely timed inputs.

Other work has focused on non-linear interactions between tuft dendrites and the soma [23–27]. In contrast, we investigated dendritic integration on a finer spatial scale—within subtrees of the dendritic tuft. Notwithstanding, we observed for all but one proximal-distal branch pair that additive feedback was sufficient to describe the somatic response. In contrast, previous work found a multiplicative coupling between the soma and the tuft [25], i.e. distal depolarizing current injections increased the gain and reduced the rheobase of the firing rate-current response at the soma. However, the underlying mechanism relied on backpropagating action potentials that haven’t been triggered in our study. We have shown that feedback between the proximal apical dendrites and the tuft occurs also in the sub-threshold regime, and this coupling is additive in most cases.

We hypothesize that this additive coupling between tuft dendrites serves as a gating mechanism similar to the proximal-distal coupling. Weakly stimulated dendritic branches in the tuft that would otherwise fail to detect individual input patterns with local membrane potentials sub-threshold to the non-linearities combine the collective evidence resulting in robust pattern recognition.

### Outlook

We investigated dendritic integration properties of branches that each receive only a single synaptic input. Hence, the subunit non-linearities are one-dimensional functions of the synaptic input strength. It has been recently shown that pairs of synaptic inputs to the same branch in the basal dendrites are not purely summed but also processed non-linearly in a location dependent manner [15, 18]. Conceptually, the two-layer model proposed here can be augmented to incorporate this intra-branch integration effects. Moreover, the theoretical framework based on iso-response methods can be applied to identify all non-linearities involved.

We focused on spatial integration properties of tuft dendrites, where pairs of synapses are activated simultaneously. A complete description of dendritic integration in pyramidal neurons has to incorporate the experimentally observed temporal coupling between the proximal dendritic compartment, including the basal dendrites, soma and apical obliques, and the distal tuft [23–27]. We expect that the two-layer model can be incorporated into this two-compartment schema.

The theoretical framework presented here can be used to identify two-layer models without the necessity of additional information beyond the stimuli and the responses and without extensive datasets. Overall, our findings support the view that the information processing capabilities of pyramidal neurons depend sensitively on the spatial distribution of synaptic inputs. This conclusion has been reached using model neurons. The proposed iso-response techniques could, however, also be integrated into multi-electrode or photo-stimulation paradigms to directly reveal the dendritic computations of biological neurons.

## Materials and methods

### The detailed compartmental model

The detailed compartmental model [28] was developed by Poirazi et al. [6] in the NEURON simulation environment. The model is well suited for our analysis because it was tested to replicate the regenerative events observed in the dendrites of pyramidal cells. The cell is a reconstructed CA1 pyramidal neuron (Fig 1A) and includes various active and passive membrane mechanisms known to be present in CA1 pyramidal cells, such as sodium and potassium currents, A-type potassium currents, m-type potassium currents, a hyperpolarization-activated h-current, voltage-dependent calcium currents, and Ca^2+^-dependent potassium currents. The densities and distributions of these currents are based on published data.

Single synaptic events were triggered simultaneously in pairs of dendritic branches (Fig 1A and 1B). Each synaptic input consisted of an NMDA and an AMPA-type conductance with a ratio of their peak values of 2.5. Synapses were located at the centers of dendritic branches. For purely passive dendrites the NMDA conductances induced highly stereotypical supralinear somatic responses (S10 Fig).

The peak conductances were varied between zero and a maximum value. The maximum value was chosen for each synaptic input such that the strongest paired inputs generated a somatic action potential. Maximum values of NMDA conductances varied between 120 and 400 nS. Assuming a maximum conductance of 4 nS for single synapses [8, 29–31] this corresponds to at most 30-100 synapses per branch—roughly the same input range as studied in related work [11] with at most 20-60 synapses per branch.

For the experiments in Fig 7A we blocked L-type and T-type calcium channels with a high threshold for activation, an R-type calcium channel with a medium threshold for activation, an A-type potassium current, and a hyperpolarization-activated cation current (*I*_*h*_). For computations based on firing rates (Fig 7B) the synaptic conductance are chosen such that for maximum paired branch stimulations (i.e. synaptic Poisson inputs at 40 Hz) the neuronal response (i.e. the mean firing rate during a stimulation duration of 500 ms) is roughly in the same range as the input rates, i.e. with a mean of 40 Hz and a standard deviation of 25 Hz (For some branches the somatic firing rate saturates below 40 Hz resulting in smaller maximum responses).

### Iso-response methods

We assume that the gradient of the response *r* = *f*_3_(*f*_1_(*s*_1_) + *f*_2_(*s*_2_)) in terms of the inputs *s*_1_ and *s*_2_ exists and is non-zero in the whole synaptic input regime. This is reasonable because the somatic voltage depolarization is a strictly monotonic function of the synaptic input strengths. Under this condition, all stimuli that cause the same response form a one dimensional “iso-response curve” in the two dimensional input space (for details see [14]). Each response value corresponds to a unique iso-response curve (Fig 1B and 1C) and each pair of *s*_1_ and *s*_2_ is an element of an iso-response curve.

To estimate iso-response curves a cubic-spline fit of the response function *r*(*s*_1_, *s*_2_) was generated. For a specific response value *r*_*i*_ the corresponding iso-response curve was found by solving *r*(*s*_1_, *s*_2_) − *r*_*i*_ = 0 using root finding algorithms. The resulting curves were both stored as a function of *s*_1_ and as a function of *s*_2_. Fig 2A–2C shows examples of iso-response curves for different combinations of supralinear and sublinear subunit non-linearities.

In this simulation study, we measured three iso-response curves

$$\begin{array}{c} \begin{array}{cc} & L:s_{1}\mapsto s_{2}=L\left(s_{1}\right)\\ & H:s_{1}\mapsto s_{2}=H\left(s_{1}\right)\\ & T:s_{1}\mapsto s_{2}=T\left(s_{1}\right). \end{array} \end{array}$$ (1)

Two curves, *L* and *H*, were chosen at a low and high value of *r* for the identification of the subunit non-linearities and a third test curve, *T*, was chosen at a response value right below the threshold for action potential generation. The test iso-response curve was at least half as long as *L* or *H*.

### Identification of two-layer models without feedback

In the following a relation between iso-response curves and the subunit functions of a conventional two-layer model will be derived. In general, one iso-response curve is not sufficient to identify the subunit non-linearities. However, we will show that two iso-response curves can be used to identify *f*_1_ and *f*_2_ up to an affine transformation. Furthermore, a third iso-response curve can be used to indicate whether *r* can be described by a two-layer model within a certain input regime.

Assume *s*_1_ and *s*_2_ = *H*(*s*_1_). The gradient of *r* in the direction of the iso-response curve *H* is zero and

$$\begin{array}{c} \frac{\partial f_{1}\left(s_{1}\right)}{\partial s_{1}}+\frac{\partial f_{2}\left(s_{2}\right)}{\partial s_{2}}\frac{\partial H(s_{1})}{\partial s_{1}}=0. \end{array}$$ (2)

If *s*_1_ and $s_{2}^{\prime }=L\left(s_{1}\right)$ denote a second point on the iso-response curve *L* then the ratio of the derivatives of *f*_2_ at both points is given by

$$\begin{array}{c} \begin{array}{cc} & \frac{\frac{\partial f_{2}\left(s_{2}^{\prime }\right)}{\partial s_{2}^{\prime }}}{\frac{\partial f_{2}\left(s_{2}\right)}{\partial s_{2}}}=\frac{\frac{\partial H(s_{1})}{\partial s_{1}}}{\frac{\partial L(s_{1})}{\partial s_{1}}}. \end{array} \end{array}$$ (3)

Analogous, for two points with identical *s*_2_ but different *s*_1_ = *H*^−1^(*s*_2_) and $s_{1}^{\prime }=L^{-1}\left(s_{2}\right)$ follows

$$\begin{array}{c} \begin{array}{cc} & \frac{\frac{\partial f_{1}\left(s_{1}^{\prime }\right)}{\partial s_{1}^{\prime }}}{\frac{\partial f_{1}\left(s_{1}\right)}{\partial s_{1}}}=\frac{\frac{\partial H^{-1}\left(s_{2}\right)}{\partial s_{2}}}{\frac{\partial L^{-1}\left(s_{2}\right)}{\partial s_{2}}}, \end{array} \end{array}$$ (4)

where *L*^−1^ and *H*^−1^ denote the inverse functions of *L* and *H*, respectively. The same relations are valid if the partial derivatives are replaced by finite differences (Fig 2E).

Given an odd number of *n* points that form a stairway

$$\begin{array}{c} \{\left(s_{1}^{\left(1\right)},s_{2}^{\left(1\right)}\right),\left(s_{1}^{\left(1\right)},s_{2}^{\left(2\right)}\right),\left(s_{1}^{\left(2\right)},s_{2}^{\left(2\right)}\right),\left(s_{1}^{\left(2\right)},s_{2}^{\left(3\right)}\right),...,\left(s_{1}^{\left(n/2+1/2\right)},s_{2}^{\left(n/2+1/2\right)}\right)\}, \end{array}$$ (5)

and lie on the two iso-response curves in alternating order with

$$\begin{array}{c} s_{2}^{\left(i\right)}=H\left(s_{1}^{\left(i\right)}\right)\;\;\;\text{and}\;\;\;s_{2}^{\left(j+1\right)}=L\left(s_{1}^{\left(j\right)}\right) \end{array}$$ (6)

these relations constrain the subunit functions *f*_1_ and *f*_2_ at the points $s_{1}^{\left(i\right)}$ and $s_{2}^{\left(j\right)}$ up to an affine transformation that can be absorbed into *f*_3_ (Fig 2F and 2G). For arbitrary points that do not form a stairway, the subunit non-linearities can be expanded in basis-functions (see S1 Methods). In this case all iso-response curves of the two-layer model can be predicted within the range of input values used for the identification of the subunit non-linearities.

We call two iso-response curves *identical* if they contain the same points in the input space irrespective of their associated response values. Then, the predicted iso-response curves and the observed iso-response curves are *identical* if and only if the responses to the stimuli on these iso-response curves can be described by a two-layer model. The “if” part follows from the fact that if the observed responses and the two-layer responses are the same then so are their iso-response curves. The “only if” part follows from the implication that if the observed iso-response curves and the iso-response curves of the two-layer model are *identical* then there always exists another two-layer model with a final non-linearity such that the resulting predicted responses match the observed ones.

Point-neuron models implement a weighted linear summation of their inputs, i.e. *r*(*s*_1_, *s*_2_) = *f*_3_(*w*_1_ *s*_1_ + *w*_2_ *s*_2_). The weights *w*_1_ and *w*_2_ were set to minimize the mean squared error between the predicted and the observed responses on the two iso-response curves *L* and *H*. For a full reconstruction of the point-neuron model and the two-layer model the measurement of *f*_3_ is required, e.g., on the line *s*_1_ = *s*_2_. The method was successfully tested on iso-response curves generated by artificial two-layer models (S1 and S2 Figs).

### Identification of two-layer models with feedback

To find the subunit non-linearities *f*_1_ and *f*_2_ of a two-layer model with feedback, we analyze a hypothetical two-layer model without feedback that has the same subunit non-linearities *f*_1_ and *f*_2_. The iso-response curve *H* of this hypothetical two-layer model without feedback can be obtained from an iso-response curve of a two-layer models with feedback, denoted as *H*_fb_, with the transformation

$$\begin{array}{ccc} H(s_{1}) & = & \Delta g_{2}^{\text{mult}}H_{\text{fb}}(\frac{s_{1}-\Delta g_{1}^{\text{add}}}{\Delta g_{1}^{\text{mult}}})+\Delta g_{2}^{\text{add}}\\ \Delta g_{1/2}^{\text{add}} & = & g_{1/2}^{\text{add}}\left(r_{H}\right)-g_{1/2}^{\text{add}}\left(r_{L}\right)\\ \Delta g_{1/2}^{\text{mult}} & = & g_{1/2}^{\text{mult}}\left(r_{H}\right)/g_{1/2}^{\text{mult}}\left(r_{L}\right), \end{array}$$ (7)

where *r*_*L*_ and *r*_*H*_ denote the response values on the iso-response curves *L* and *H*, respectively. This equation can be inserted into Eqs 3 and 4. We applied a brute-force approach to find the constant optimal values of the feedback functions on the iso-response curves *H* and *T*. Feedback values were limited to a range such that the transformed test iso-response curves (of the hypothetical two-layer model without feedback) were at least half as long as each of the iso-response curves used for training. The method was successfully tested on iso-response curves generated by artificial two-layer models (S3 and S4 Figs).

### Error measure

The error of the optimized two-layer model was tested on the third iso-response curve *T*. Three error measures were applied. First, for two-layer models without feedback (Fig 3) *f*_3_ can be reconstructed and the performance can be computed as the response variance on the test iso-response curve *T* normalized by the response variance of the training data

$$\begin{array}{c} \sigma_{r}^{2}=\frac{1}{K}\sum_{k=0}^{K-1}\frac{{\left(\bar{r}-r_{k}\right)}^{2}}{{\left(r_{H}-r_{L}\right)}^{2}}. \end{array}$$ (8)

Here, $\bar{r}$ denotes the mean response values of *K* samples on the test iso-response curve *T*, and *r*_*H*_ and *r*_*L*_ denote the response values on the iso-response curves *H* and *L*, respectively.

Secondly, for two-layer models with feedback (Fig 5) the performance can be similarly assessed for the summed output of the subunit non-linearities *m* = *f*_1_(*s*_1_) + *f*_2_(*s*_2_) with

$$\begin{array}{c} \sigma_{m}^{2}=\frac{1}{K}\sum_{k=0}^{K-1}\frac{{\left(\bar{m}-m_{k}\right)}^{2}}{{\left(m_{H}-m_{L}\right)}^{2}}, \end{array}$$ (9)

where $\bar{m}$ denotes the mean value of *K* samples on the test iso-response curve *T*, and *m*_*H*_ and *m*_*L*_ denote the value of *m* on the two iso-response curves *H* and *L*, respectively.

Finally, we also calculate for two-layer models without feedback the optimized mean square error between the predictions and the responses on the test iso-response curve *T* normalized by the response variance of the training data

$$\begin{array}{c} \text{MSE}\left(r\right)=\frac{1}{K}\sum_{k=0}^{K-1}\frac{{\left(r^{T}-r_{k}\right)}^{2}}{{\left(r_{H}-r_{L}\right)}^{2}}, \end{array}$$ (10)

where *r*^*T*^ denotes the observed response on the test iso-response curve. In general, *r*^*T*^ differs from the mean predicted response $\bar{r}$ as used for the calculation of $\sigma_{r}^{2}$.

### Testing subunit independence

For the feedforward two-layer model, subunit independence can be tested (Fig 6) if the gradient of *r* is given for four points *A*, *B*, *C* and *D* in the input space, where $A=(s_{1}^{A},s_{2}^{A})$, such that $s_{1}^{A}=s_{1}^{B}$, $s_{1}^{C}=s_{1}^{D}$, $s_{2}^{B}=s_{2}^{C}$ and $s_{2}^{A}=s_{2}^{D}$. The angle *α* between the *s*_1_-axis and a gradient-vector is given by

$$\begin{array}{c} \text{tan}\left(\alpha \left(s_{1},s_{2}\right)\right)=\frac{\frac{df_{2}\left(s_{2}\right)}{ds_{2}}}{\frac{df_{1}\left(s_{1}\right)}{ds_{2}}}. \end{array}$$ (11)

It follows

$$\begin{array}{c} \text{tan}\left(\alpha_{D}\right)=\frac{\text{tan}\left(\alpha_{A}\right)\cdot \text{tan}\left(\alpha_{C}\right)}{\text{tan}(\alpha_{B})}. \end{array}$$ (12)

This relation can be used to predict *α*_*D*_ from the three other angles. A non-zero prediction error indicates that the response cannot be described by a two-layer model.

In Fig 6B and 6F we measured the gradients on two straight lines through the input space. First, we kept *s*_2_ at a constant low value and varied *s*_1_ over the whole range ∇*r*(*s*_1_, *s*_2_ = *const*). Then, we kept *s*_1_ at a constant low value and varied *s*_2_ over the whole input range ∇*r*(*s*_1_ = *const*, *s*_2_). From these two straight lines it is possible to predict all gradients of the input space and compare them to the actual measured gradients. Keeping the constant values *s*_1_ and *s*_2_ at a low level has the benefit that synaptic integration was alway found to be linear and can be described by a two-layer model without feedback. Therefore, it is possible to determine in what input regime feedback effects occur.

## Supporting information

S1 Fig Reconstruction of a feedforward two-layer model. (A) Response function with three iso-response curves. (B) Iso-response curves from A. (C)—(E) Reconstructed subunit functions (black lines) and theoretical subunit functions (dotted red lines) used to generate the response. (F) Iso-response curves predicted by the reconstructed two-layer model (black lines) and measured iso-response curves (colored lines). (G) Difference between the predicted response and the actual response (mean absolute difference 0.0096 mV).
(TIF)

S2 Fig Incorrect reconstruction of a two-layer model with additive and multiplicative feedback from iso-response curves generated by a feedforward two-layer model (S4 Fig). (A) Incorrectly assumed multiplicative feedback increases the error on a test iso-response curve with increasing feedback strength. (B) Error of reconstructed two-layer models in dependence on the summed feedback strengths. (C) and (D) same as A and B for additive feedback. In (C) and (D) feedback is an additive factor to synaptic input whereas in (A) and (B) the difference between two multiplicative factors is shown.
(TIF)

S3 Fig Reconstruction of a two-layer model with positive additive feedback. (A) Response function of a two-layer model with step-shaped additive feedback between the yellow and the blue iso-response curve. (B) Reconstruction of a feedforward two-layer model results in a high prediction error (up to 3.6 mV). (C) Error of two-layer models with different additive feedback strengths. (D) The minimum error is obtained for the correct additive feedback strength. However, other models might also show a low prediction error on the test iso-response curve. (E) and (F) Reconstruction of two-layer models with multiplicative feedback results in prediction errors larger than zero.
(TIF)

S4 Fig Reconstruction of a two-layer model with positive multiplicative feedback. (A) Response function of a two-layer model with step-shaped multiplicative feedback between the yellow and the blue iso-response curve. (B) Reconstruction of a feedforward two-layer model results in a high prediction error (up to 2.65 mV). (C) Error of two-layer models with different multiplicative feedback strengths. (D) The minimum error is obtained for the correct multiplicative feedback strength. (E) and (F) Reconstruction of two-layer models with additive feedback results in prediction errors larger than zero.
(TIF)

S5 Fig Test of the iso-response method for a pair of proximal dendritic branches. (A) The somatic response to single branch stimulations (top panels, as estimated in S10 Fig) and the response predicted by a feedforward two-layer model (middle panels) are shown for passive dendrites. The subunit non-linearities of the two-layer model (bottom panels) are identified by the iso-response method. Colors indicate results for different levels of the AMPA/NMDA ratio that controls the strength of the dendritic supralinearity (see [32]). The final function *f*_3_ of the two-layer model is nearly linear and the somatic responses follow closely the reconstructed subunit non-linearities. (B) Results as shown in A but for active dendrites. (C) The reconstructed subunit non-linearities are similar to the measured somatic responses for nearly all P-P and T-P branch pairs (except for P14-P15, P15-P17 and P16-P17 associated with the three outliers in the left most panel). The pairwise Pearson’s correlation coefficients range from 0.96 to 1 with a mean of 0.99. For tuft dendrites the somatic responses to single branch stimulations often differ from the reconstructed subunit non-linearities with pairwise Pearson’s correlation coefficients ranging from 0.82 to 1 with a mean of 0.96.
(TIF)

S6 Fig Classes of subunit functions. (A) Relationship between the location of dendritic branches and the reconstructed subunit non-linearities indicated by the color code defined in B and D (right panels). (B) Normalized subunit non-linearities for pairs of dendritic branches in the tuft (subtree T22-T27) aligned to the onset of their non-linearities (left panel). The normalization involves a vertical and horizontal scaling and shift of each curve. The color matrix (right panel) indicates the shapes of the subunit functions (left panel). The row index shows the dendritic branch for which the subunit non-linearity is reconstructed. The column index indicates the second branch of a stimulated branch pair. The branches T22 and T24 (subunit non-linearities shown in light green) have a diameter of 1.1*μ*m and the branches T23, T25, T26 and T27 have a diameter of 0.8*μ*m (subunit non-linearities shown in dark green) resulting in different input impedances (see S7 Fig). (C) Normalized subunit non-linearities for T-P branch pairs aligned to the onset of their non-linearities (left panel) as in B. The subunit non-linearities resemble those for P-P and T-T branch pairs but incorporate additional non-linearities between the outputs of the dendritic branches and the site of their summation. For proximal branches (left panel) the subunit non-linearities are steep when the second stimulated branch is located in subtree T2 and flat when the second stimulated branch is located in subtree T3 (see S15 Fig for details). (D) Normalized subunit non-linearities for pairs of proximal apical branches aligned to the onset of their non-linearities (left panel) as in B. The non-linearities can be divided into three classes of approximately linear, weakly supralinear and strongly supralinear functions according to their input impedance (see S7 Fig). The color matrix (right panel) indicates the shapes of the subunit functions (left panel). The row index shows the dendritic branch for which the subunit non-linearity is reconstructed. The column index indicates the second branch of a stimulated branch pair.
(TIF)

S7 Fig Somatic and dendritic responses to single branch stimulations. (A) Response (local depolarizations averaged over time) in the dendritic tree (rows of the matrix) in response to single branch stimulations (columns of the matrix). (B) Attenuations of depolarizations from the sites of synaptic stimulations to the soma.
(TIF)

S8 Fig Comparison of error measures for point-neuron models and feed forward two-layer model. Results from Fig 3 for two different types of error measure (see methods) are qualitatively similar.
(TIF)

S9 Fig Performance improvement of the feedforward two-layer model for blocked active ion channels in the tuft. (A) Performance for 25 branch pairs. Voltage-gated calcium currents (*I*_*Ca*_), an A-type potassium current (*I*_*A*_), and a hyperpolarization-activated cation current (*I*_*h*_) are blocked individually in the tuft. The block of each of these currents improves the performance (except for T33-T35). (B) Response (somatic voltage averaged over time) on the test iso-response curves for the three blocking scenarios and control. Only the block of *I*_*h*_ significantly decreases the response values and potentially reduces the dendritic non-linearities related to the voltage dependence of NMDA channels.
(TIF)

S10 Fig Somatic depolarizations for single branch stimulations. (A) Somatic depolarizations averaged over 50 ms in response to stimulations of individual dendritic branches. For passive dendrites with all but the leak and the synaptic conductances blocked the responses are shown for tuft branches (red line), proximal apical branches (blue lines) and the tuft (solid black lines). The black dashed lines are extrapolations of the linear responses to small synaptic input conductances. The somatic depolarizations for active dendrites (solid gray lines) are truncated at the somatic spike threshold (vertical black lines) and scaled by a multiplicative factor to obtain the same slopes of the linear responses to small conductance values as for passive dendrites. (B) Somatic depolarizations for passive dendrites in units of the inflection point. The somatic responses are highly stereotypical with pairwise Pearson’s correlation coefficients larger than 0.99. (C) Somatic depolarizations for active dendrites in units of the inflection point obtained in B. Somatic spike thresholds are indicated by vertical black lines. The responses are less stereotypical compared to passive dendrites with pairwise Pearson’s correlation coefficients (for overlapping synaptic conductance intervals) ranging from 0.85 to 0.99 with a mean of 0.98.
(TIF)

S11 Fig Feedback strengths. Color code indicates the difference in feedback strengths between both training iso-response curves L and H for the dendritic branch selected by the row label. (A,C) Additive feedback. (B,D) Multiplicative feedback.
(TIF)

S12 Fig Performance of the feedforward two-layer model for oblique dendrites. (A) Apical dendrite flattened in 2D. Selected oblique branches are indicated by labels (O). (B) Prediction error of point-neuron models. (C) Prediction error of feedforward two-layer models.
(TIF)

S13 Fig Subunit functions for P-P branch pairs. Each panel row shows the subunit functions of all dendritic branches located in the subtrees P1, P2, P3 or the trunk, respectively. Dendritic branches are indicated by colors. Color codes shown in the right most panels apply to all panels of the respective panel row. Each panel of a panel column shows the subunit functions for all partner stimulation sites (to generate iso-response curves) located in the subtrees P1, P2, P3 or the trunk, respectively. Thus, off-diagonal panels in the panel matrix correspond to dendritic branches located in different subtrees and panels on the main diagonal to branches located within the same subtree. Subunit functions with (without) linear onsets are normalized to unit slope (a slope of five) for the initial part of the function. Colored numbers indicate the number of curves shown in the same color. Subunit functions reconstructed from branch pairs located in different subtrees are independent of the location of the partner branch within the other subtree. Subunit functions reconstructed from branch pairs located in the same subtree depend on the precise location of the summation of both subunit outputs (see S6D Fig). The strongest subunit non-linearities are observed for the dendritic branches P13 to P16 when the partner branch is either P10 or P11 (and located outside of the functional compartment). The most distal dendritic branches P14 and P15 show the strongest non-linearities. Asterisks indicate subunit non-linearities reconstructed with partner branch P12.
(TIF)

S14 Fig Subunit functions for T-T branch pairs. Each panel row shows the subunit functions of all dendritic branches located in the subtrees T2 or T3, respectively. Dendritic branches are indicated by colors. Color codes shown in the right most panels apply to all panels of the respective panel row. Each panel of a panel column shows the subunit functions for all partner stimulation sites (to generate iso-response curves) located in the subtrees T2 or T3, respectively. Subunit functions are normalized to unit slope for the initial part of the function. Colored numbers indicate the number of curves shown in the same color. Subunit functions reconstructed from branch pairs located in different subtrees are rare and can be considered as false positives. Subunit functions reconstructed from branch pairs located in the subtree T22-T27 are either weakly or strongly supralinear depending on the branch morphology (see S6B Fig). Subunit functions reconstructed from branch pairs located in the subtree T3 are mostly linear (parts of presumably extended non-linearities). In this case, putative linear feedback might be incorporated in a two-layer feedforward model.
(TIF)

S15 Fig Subunit functions for T-P branch pairs. Each panel row shows the subunit functions of all selected dendritic branches located in the subtrees P1, P2, P3, T2 or T3, respectively. Dendritic branches are indicated by colors. Color codes shown in the right most panels apply to all panels of the respective panel row. Each panel of a panel column shows the subunit functions for all partner stimulation sites (to generate iso-response curves) located in the subtrees P1, P2, P3, T2 or T3, respectively. Subunit functions with (without) linear onsets are normalized to unit slope (a slope of five) for the initial part of the function. Colored numbers indicate the number of curves shown in the same color. For all panels, the more distal the dendritic branches the earlier (smaller conductance values) and stronger is the dendritic non-linearity.
(TIF)

S1 Methods Numerical estimation of subunit non-linearities. Estimation of subunit non-linearities for input points that do not form a stairway.
(PDF)

S1 File Python notebook for iso-response analysis. The Python notebook “iso_response_analysis.ipynb” performs an iso-response analysis for a test two-layer model. It can be run with jupyter and reproduces S4–S7 Figs.
(IPYNB)
