# Supplementary figures and images for: Tuft dendrites of pyramidal neurons operate as feedback-modulated functional subunits
Source: PLoS Comput Biol. 2019 Mar 6;15(3):e1006757. doi: 10.1371/journal.pcbi.1006757 (PMC6402658; doi:10.1371/journal.pcbi.1006757)

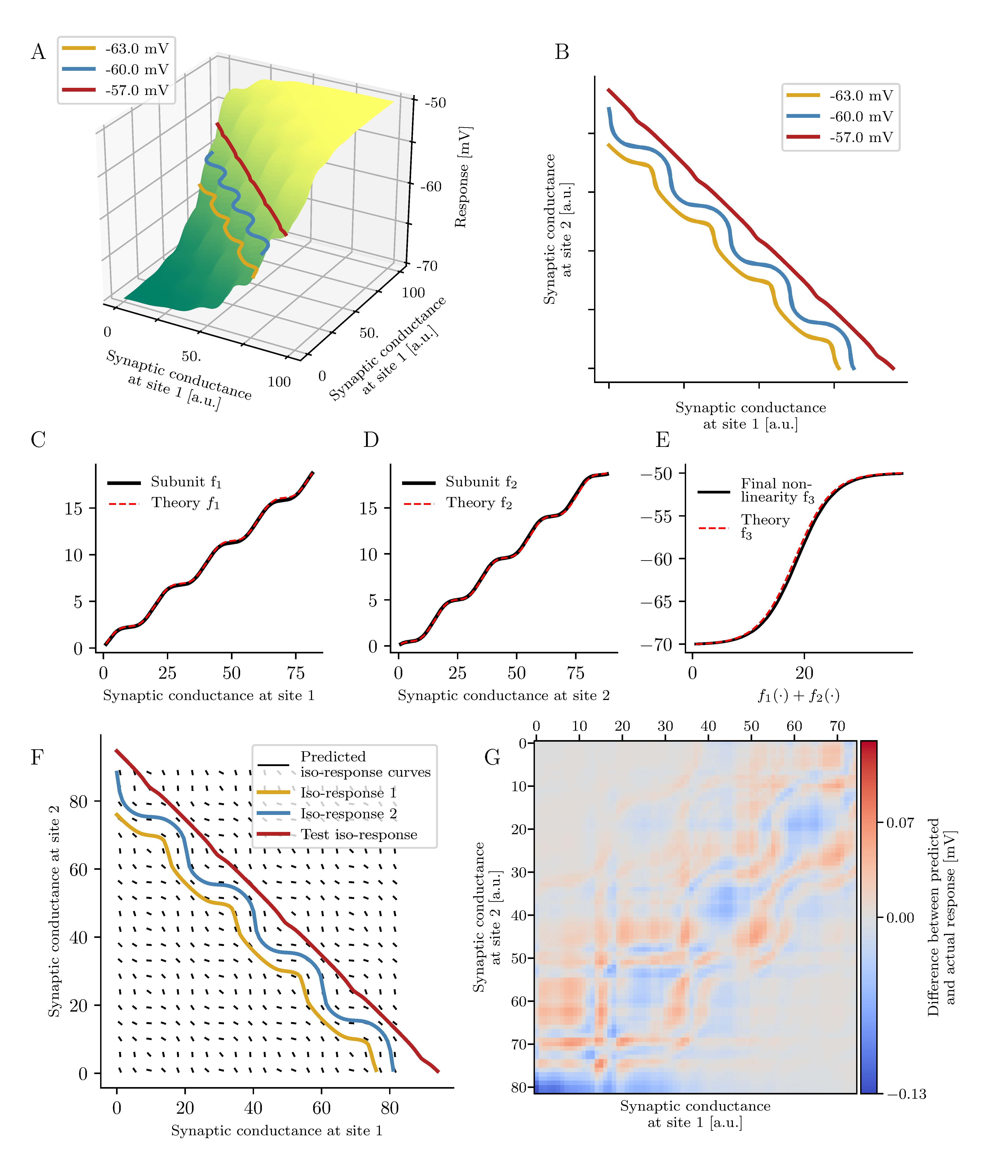

Supplement: S1 Fig — (A) Response function with three iso-response curves. (B) Iso-response curves from A. (C)—(E) Reconstructed subunit functions (black lines) and theoretical subunit functions (dotted red lines) used to generate the response. (F) Iso-response curves predicted by the reconstructed two-layer model (black lines) and measured iso-response curves (colored lines). (G) Difference between the predicted response and the actual response (mean absolute difference 0.0096 mV). (TIF) [file pcbi.1006757.s001.tif]

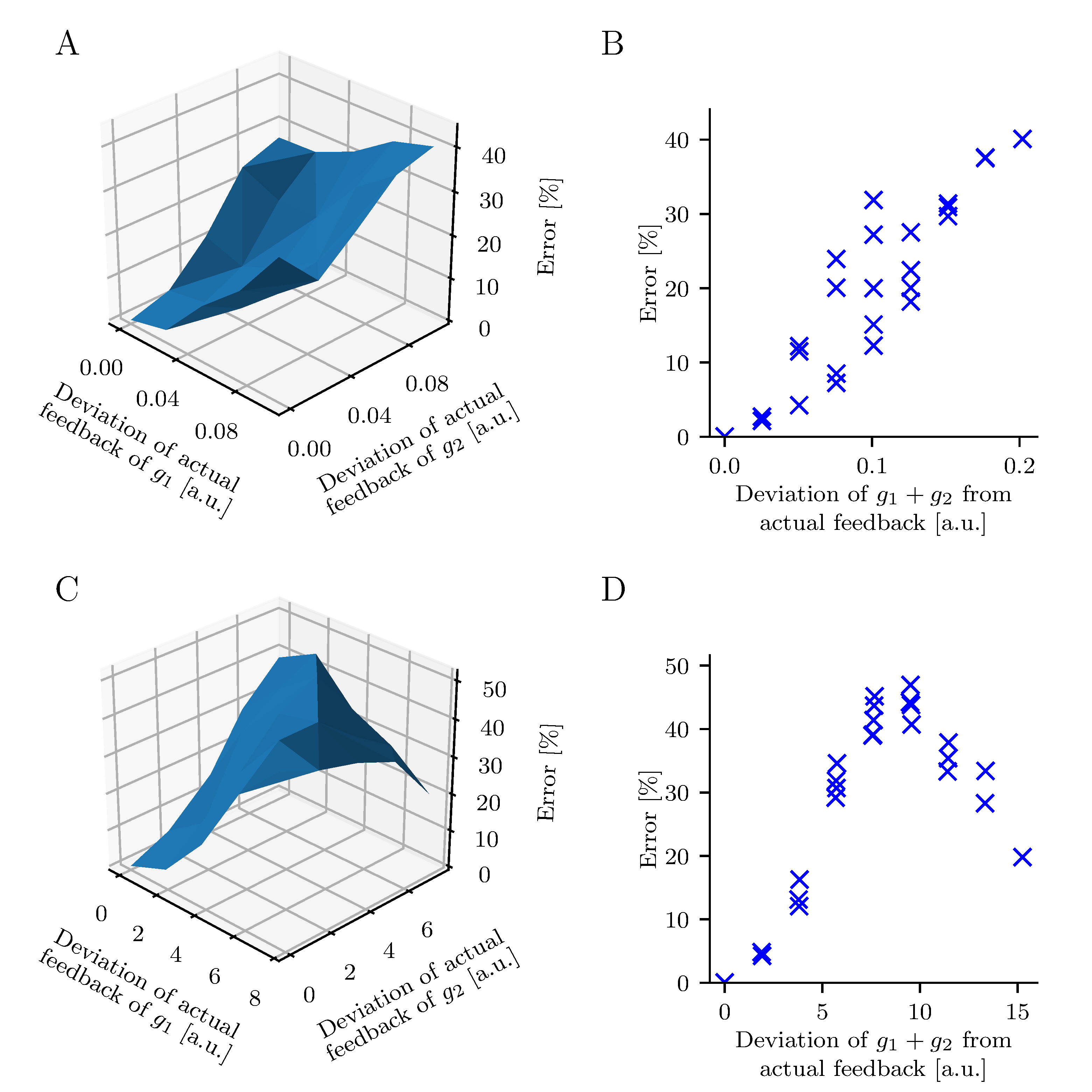

Supplement: S2 Fig — (A) Incorrectly assumed multiplicative feedback increases the error on a test iso-response curve with increasing feedback strength. (B) Error of reconstructed two-layer models in dependence on the summed feedback strengths. (C) and (D) same as A and B for additive feedback. In (C) and (D) feedback is an additive factor to synaptic input whereas in (A) and (B) the difference between two multiplicative factors is shown. (TIF) [file pcbi.1006757.s002.tif]

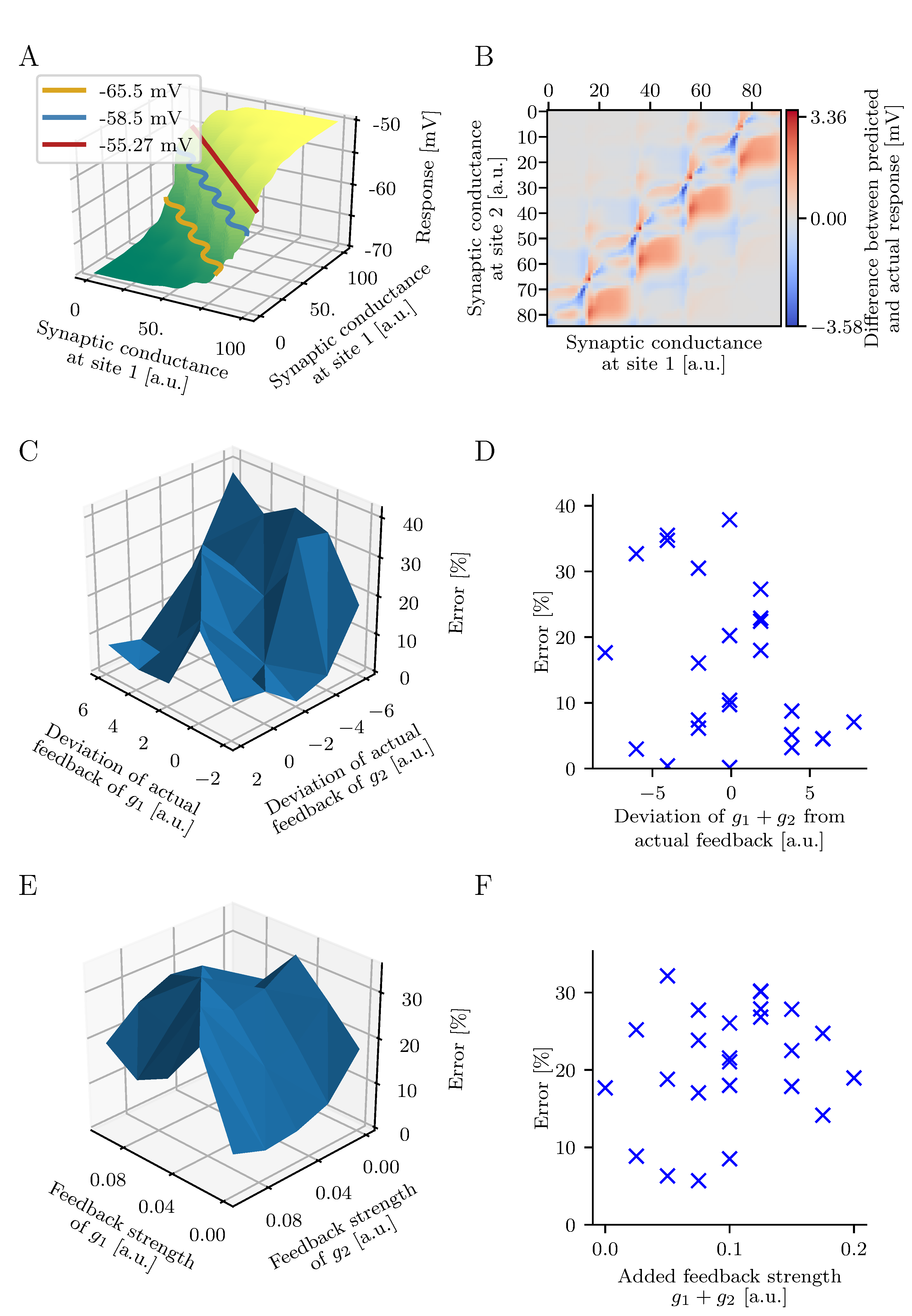

Supplement: S3 Fig — (A) Response function of a two-layer model with step-shaped additive feedback between the yellow and the blue iso-response curve. (B) Reconstruction of a feedforward two-layer model results in a high prediction error (up to 3.6 mV). (C) Error of two-layer models with different additive feedback strengths. (D) The minimum error is obtained for the correct additive feedback strength. However, other models might also show a low prediction error on the test iso-response curve. (E) and (F) Reconstruction of two-layer models with multiplicative feedback results in prediction errors larger than zero. (TIF) [file pcbi.1006757.s003.tif]

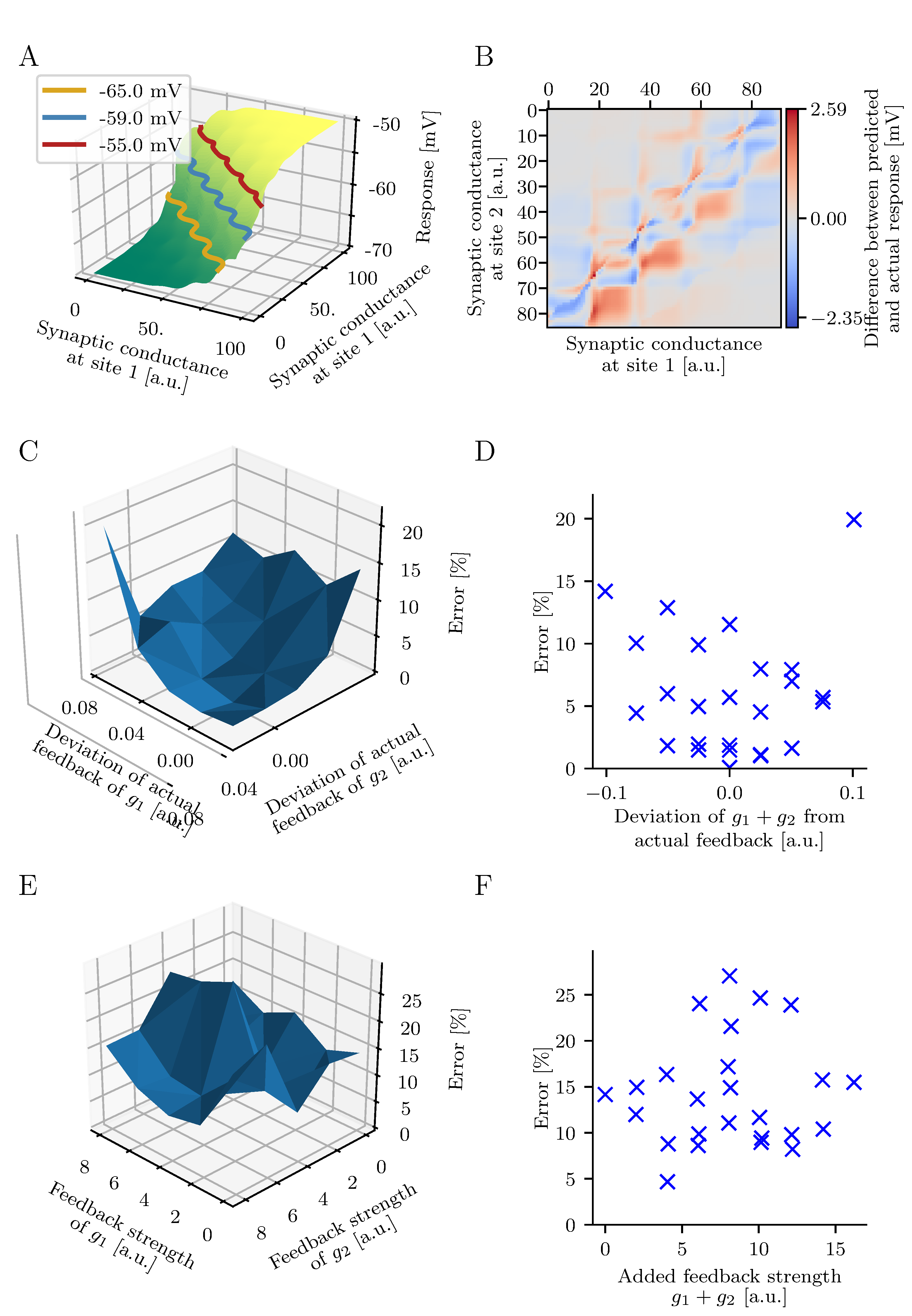

Supplement: S4 Fig — (A) Response function of a two-layer model with step-shaped multiplicative feedback between the yellow and the blue iso-response curve. (B) Reconstruction of a feedforward two-layer model results in a high prediction error (up to 2.65 mV). (C) Error of two-layer models with different multiplicative feedback strengths. (D) The minimum error is obtained for the correct multiplicative feedback strength. (E) and (F) Reconstruction of two-layer models with additive feedback results in prediction errors larger than zero. (TIF) [file pcbi.1006757.s004.tif]

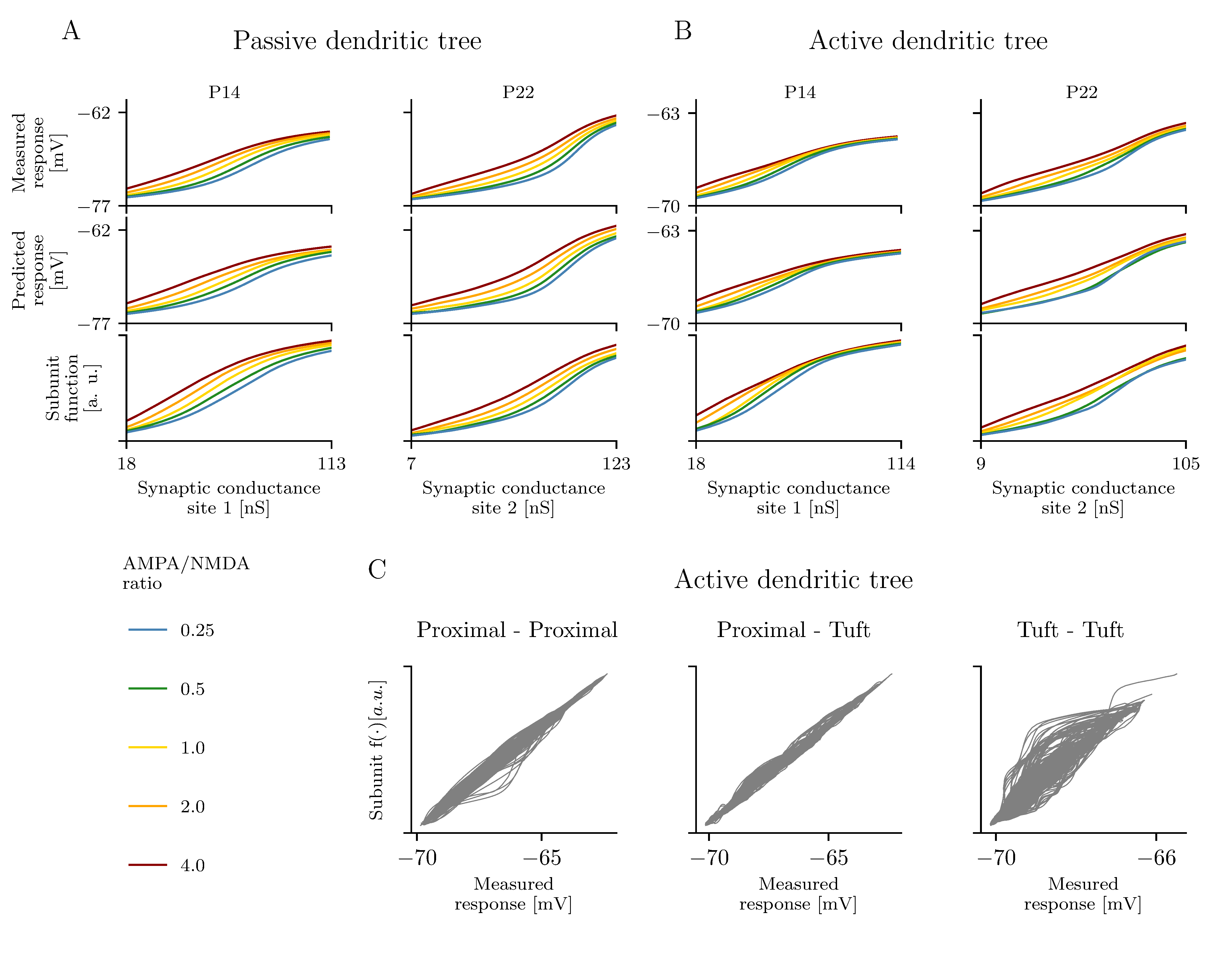

Supplement: S5 Fig — (A) The somatic response to single branch stimulations (top panels, as estimated in S10 Fig) and the response predicted by a feedforward two-layer model (middle panels) are shown for passive dendrites. The subunit non-linearities of the two-layer model (bottom panels) are identified by the iso-response method. Colors indicate results for different levels of the AMPA/NMDA ratio that controls the strength of the dendritic supralinearity (see [32]). The final function f3 of the two-layer model is nearly linear and the somatic responses follow closely the reconstructed subunit non-linearities. (B) Results as shown in A but for active dendrites. (C) The reconstructed subunit non-linearities are similar to the measured somatic responses for nearly all P-P and T-P branch pairs (except for P14-P15, P15-P17 and P16-P17 associated with the three outliers in the left most panel). The pairwise Pearson’s correlation coefficients range from 0.96 to 1 with a mean of 0.99. For tuft dendrites the somatic responses to single branch stimulations often differ from the reconstructed subunit non-linearities with pairwise Pearson’s correlation coefficients ranging from 0.82 to 1 with a mean of 0.96. (TIF) [file pcbi.1006757.s005.tif]

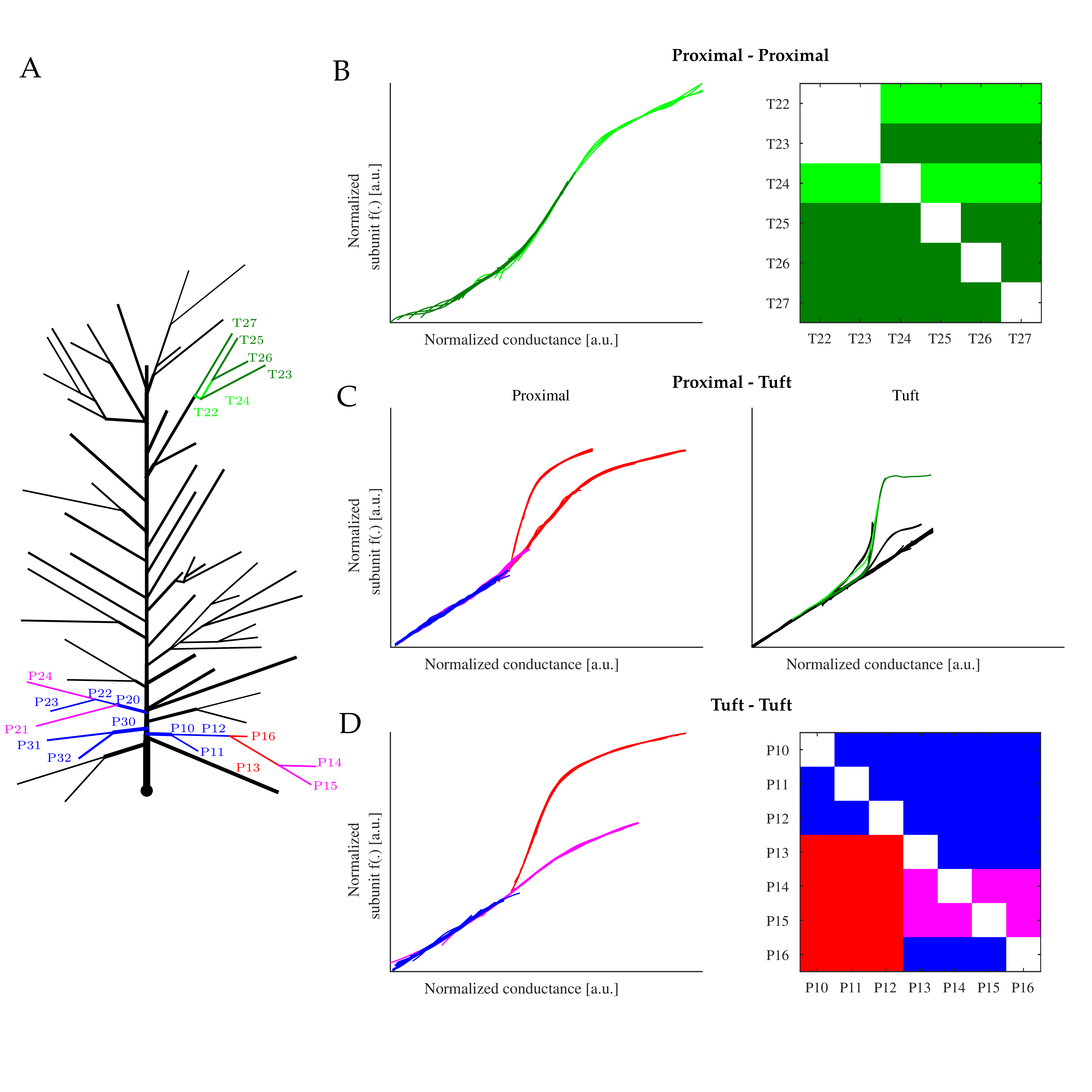

Supplement: S6 Fig — (A) Relationship between the location of dendritic branches and the reconstructed subunit non-linearities indicated by the color code defined in B and D (right panels). (B) Normalized subunit non-linearities for pairs of dendritic branches in the tuft (subtree T22-T27) aligned to the onset of their non-linearities (left panel). The normalization involves a vertical and horizontal scaling and shift of each curve. The color matrix (right panel) indicates the shapes of the subunit functions (left panel). The row index shows the dendritic branch for which the subunit non-linearity is reconstructed. The column index indicates the second branch of a stimulated branch pair. The branches T22 and T24 (subunit non-linearities shown in light green) have a diameter of 1.1μm and the branches T23, T25, T26 and T27 have a diameter of 0.8μm (subunit non-linearities shown in dark green) resulting in different input impedances (see S7 Fig). (C) Normalized subunit non-linearities for T-P branch pairs aligned to the onset of their non-linearities (left panel) as in B. The subunit non-linearities resemble those for P-P and T-T branch pairs but incorporate additional non-linearities between the outputs of the dendritic branches and the site of their summation. For proximal branches (left panel) the subunit non-linearities are steep when the second stimulated branch is located in subtree T2 and flat when the second stimulated branch is located in subtree T3 (see S15 Fig for details). (D) Normalized subunit non-linearities for pairs of proximal apical branches aligned to the onset of their non-linearities (left panel) as in B. The non-linearities can be divided into three classes of approximately linear, weakly supralinear and strongly supralinear functions according to their input impedance (see S7 Fig). The color matrix (right panel) indicates the shapes of the subunit functions (left panel). The row index shows the dendritic branch for which the subunit non-linearity is reconstructed. T [file pcbi.1006757.s006.tif]

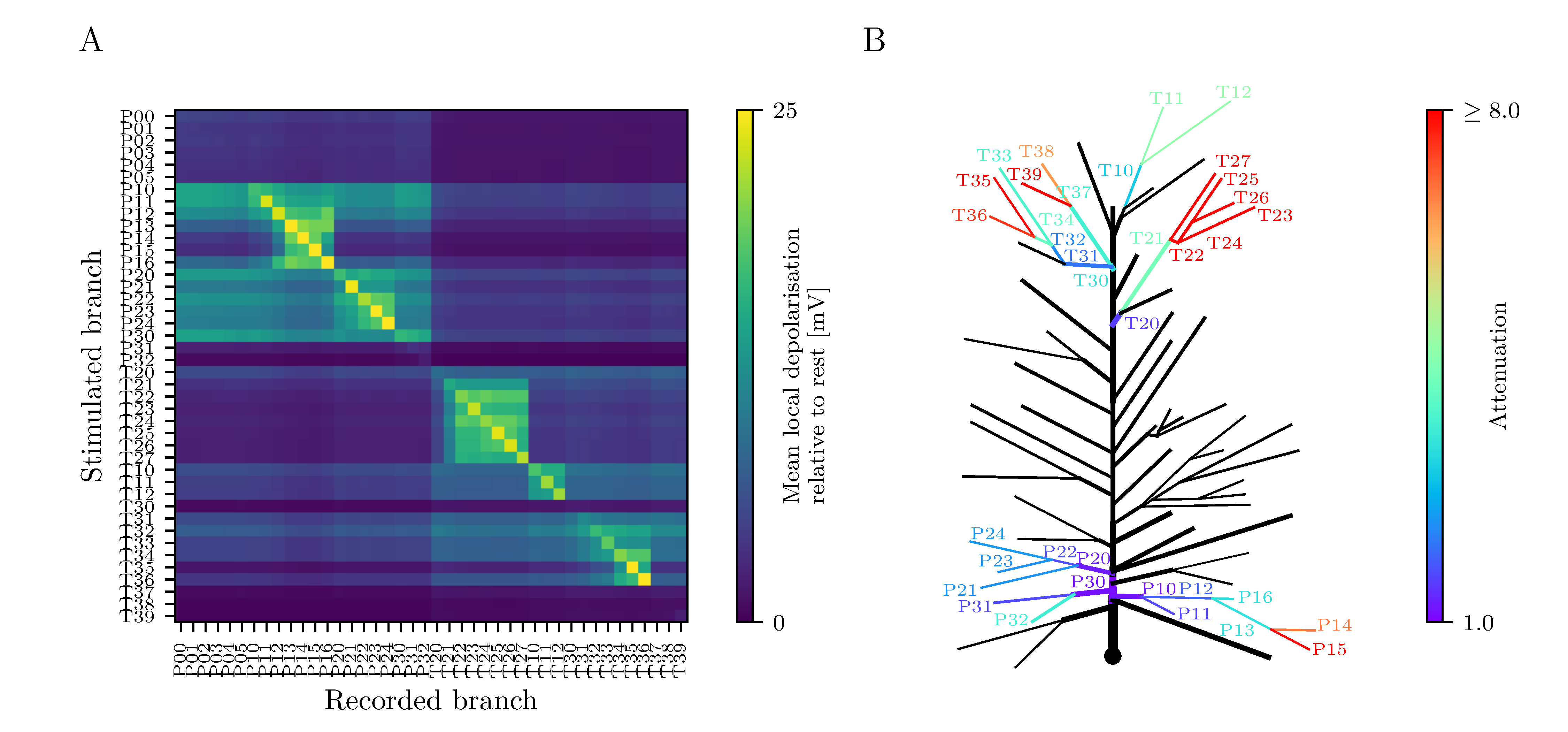

Supplement: S7 Fig — (A) Response (local depolarizations averaged over time) in the dendritic tree (rows of the matrix) in response to single branch stimulations (columns of the matrix). (B) Attenuations of depolarizations from the sites of synaptic stimulations to the soma. (TIF) [file pcbi.1006757.s007.tif]

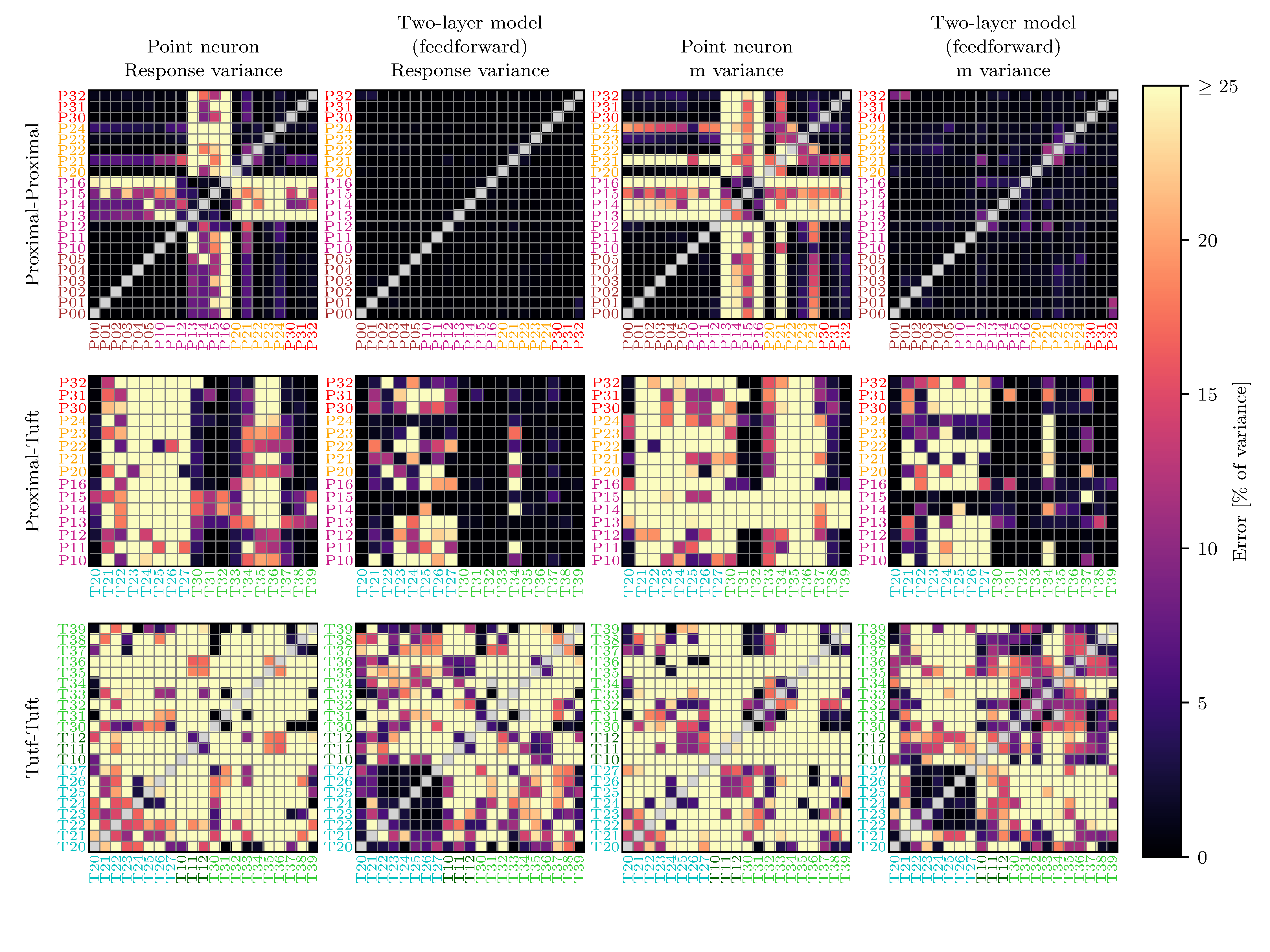

Supplement: S8 Fig — Results from Fig 3 for two different types of error measure (see methods) are qualitatively similar. (TIF) [file pcbi.1006757.s008.tif]

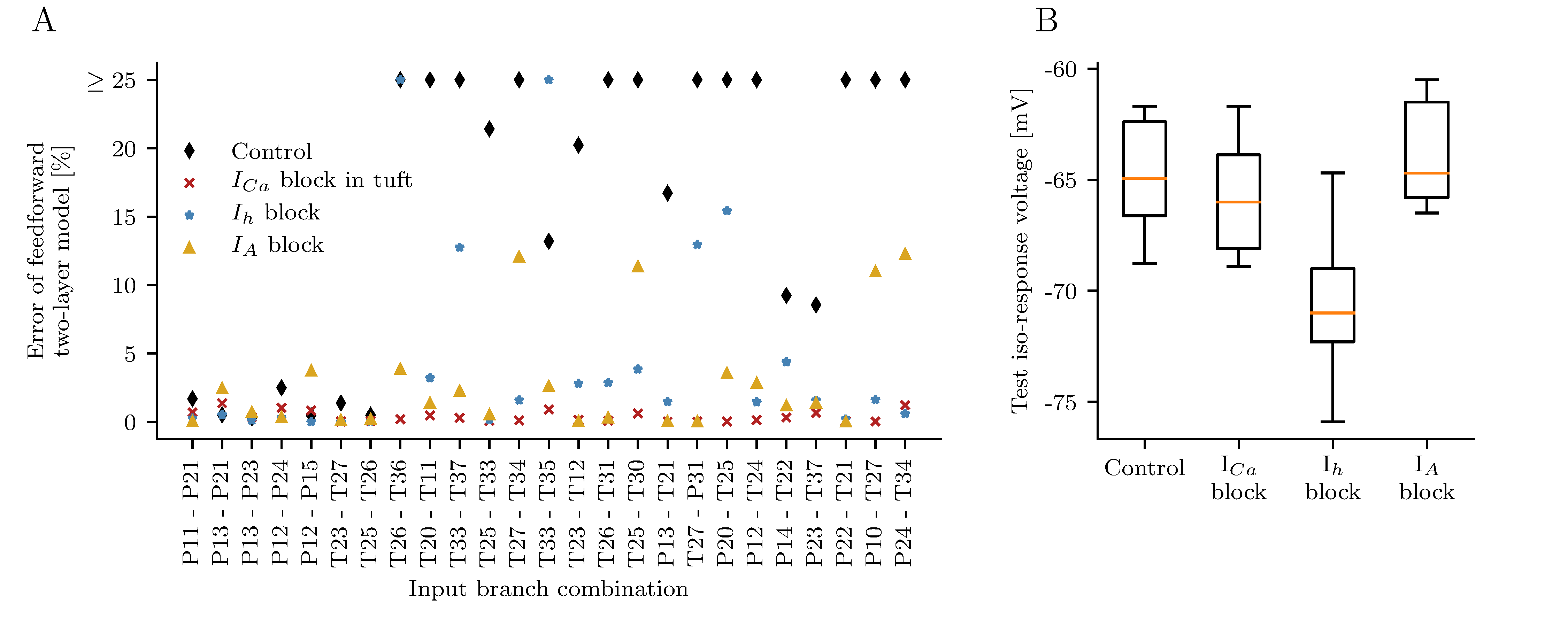

Supplement: S9 Fig — (A) Performance for 25 branch pairs. Voltage-gated calcium currents (ICa), an A-type potassium current (IA), and a hyperpolarization-activated cation current (Ih) are blocked individually in the tuft. The block of each of these currents improves the performance (except for T33-T35). (B) Response (somatic voltage averaged over time) on the test iso-response curves for the three blocking scenarios and control. Only the block of Ih significantly decreases the response values and potentially reduces the dendritic non-linearities related to the voltage dependence of NMDA channels. (TIF) [file pcbi.1006757.s009.tif]

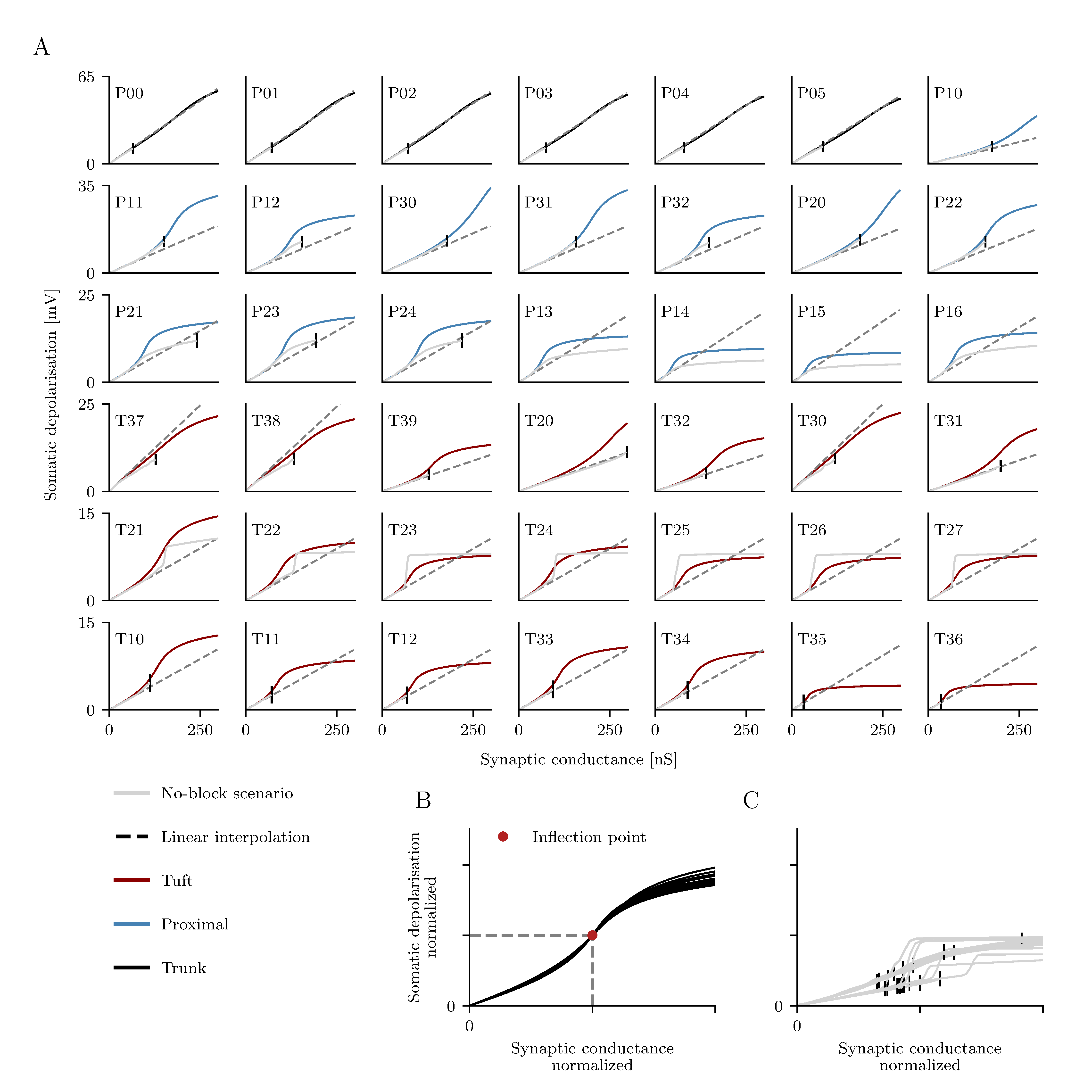

Supplement: S10 Fig — (A) Somatic depolarizations averaged over 50 ms in response to stimulations of individual dendritic branches. For passive dendrites with all but the leak and the synaptic conductances blocked the responses are shown for tuft branches (red line), proximal apical branches (blue lines) and the tuft (solid black lines). The black dashed lines are extrapolations of the linear responses to small synaptic input conductances. The somatic depolarizations for active dendrites (solid gray lines) are truncated at the somatic spike threshold (vertical black lines) and scaled by a multiplicative factor to obtain the same slopes of the linear responses to small conductance values as for passive dendrites. (B) Somatic depolarizations for passive dendrites in units of the inflection point. The somatic responses are highly stereotypical with pairwise Pearson’s correlation coefficients larger than 0.99. (C) Somatic depolarizations for active dendrites in units of the inflection point obtained in B. Somatic spike thresholds are indicated by vertical black lines. The responses are less stereotypical compared to passive dendrites with pairwise Pearson’s correlation coefficients (for overlapping synaptic conductance intervals) ranging from 0.85 to 0.99 with a mean of 0.98. (TIF) [file pcbi.1006757.s010.tif]

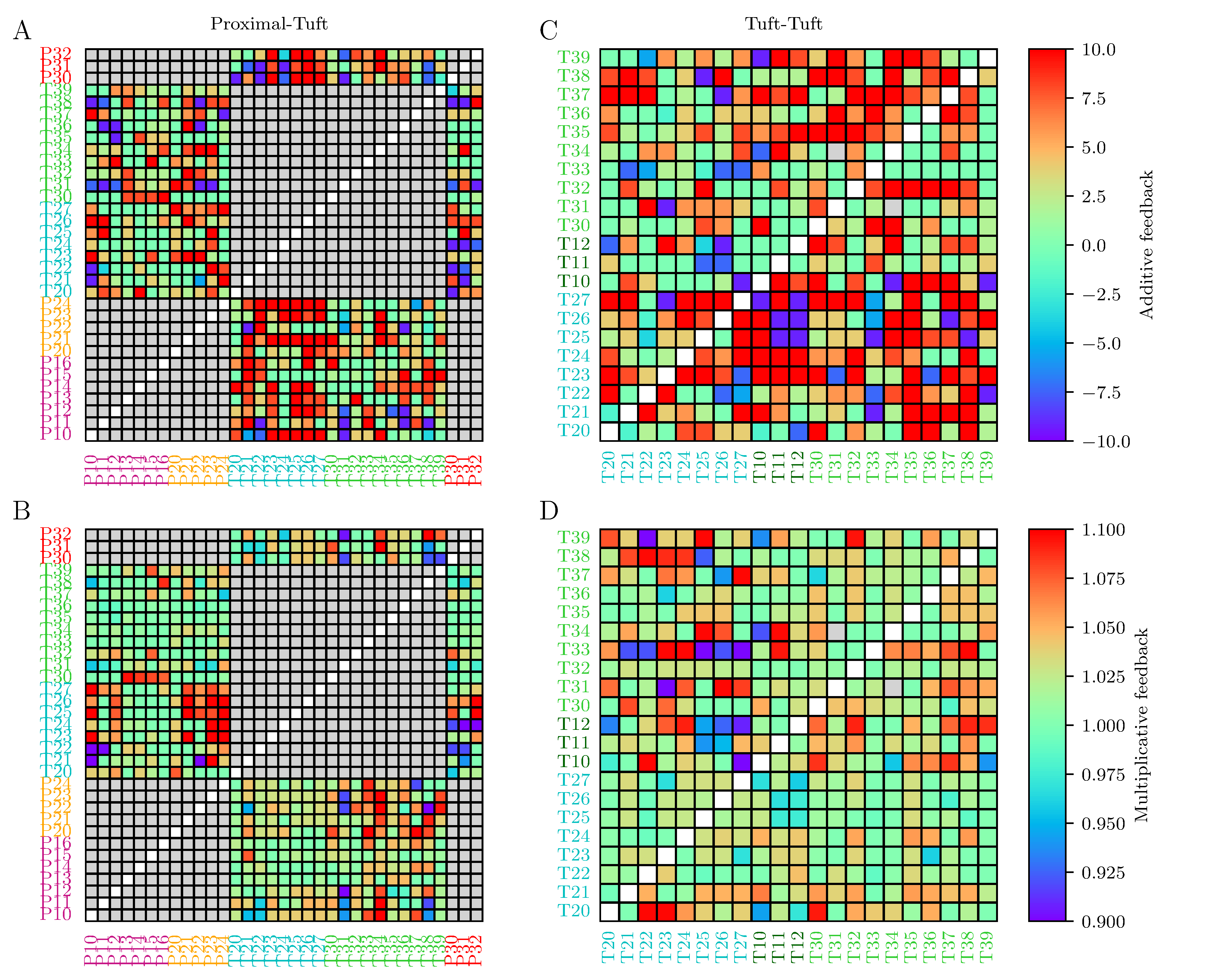

Supplement: S11 Fig — Color code indicates the difference in feedback strengths between both training iso-response curves L and H for the dendritic branch selected by the row label. (A,C) Additive feedback. (B,D) Multiplicative feedback. (TIF) [file pcbi.1006757.s011.tif]

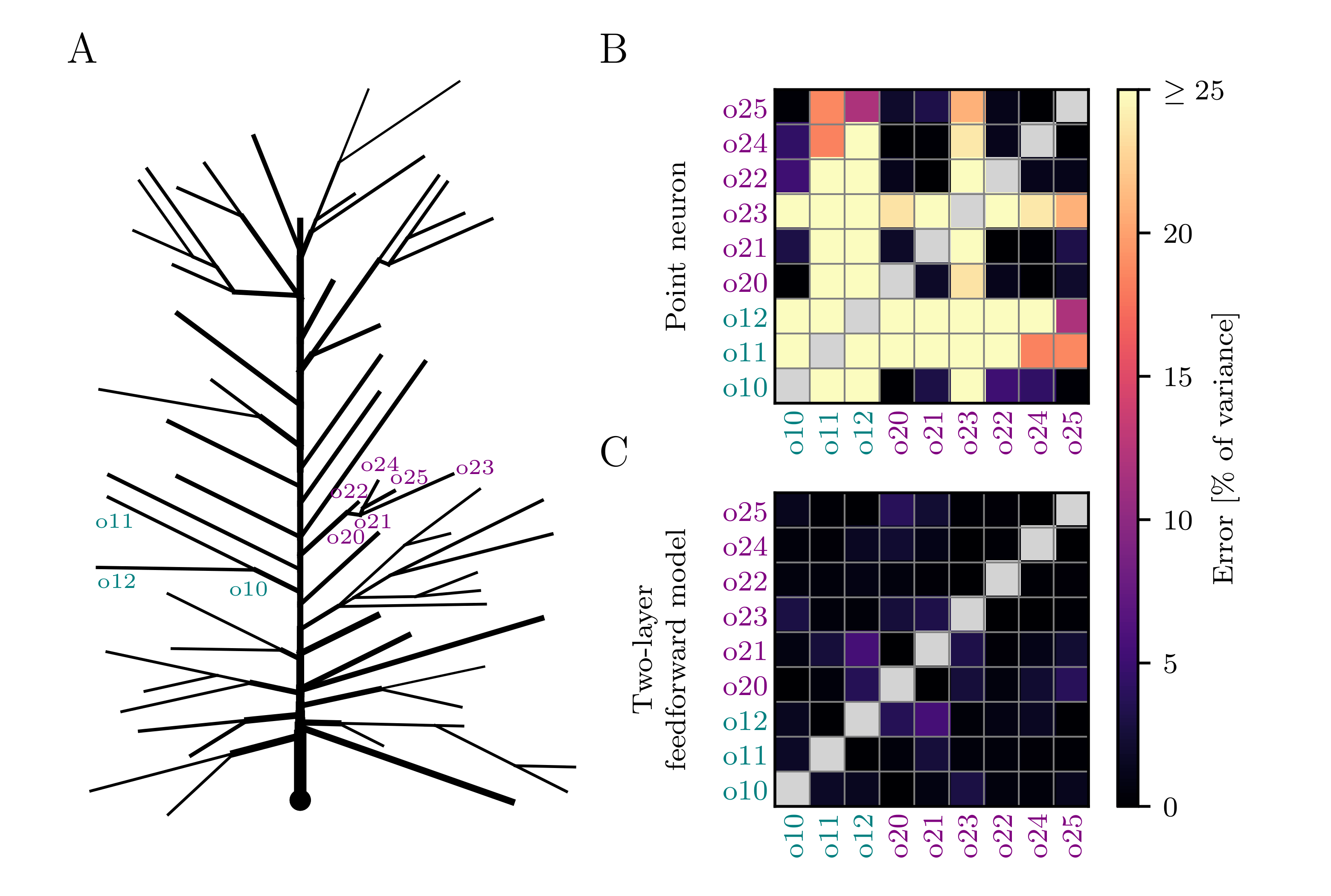

Supplement: S12 Fig — (A) Apical dendrite flattened in 2D. Selected oblique branches are indicated by labels (O). (B) Prediction error of point-neuron models. (C) Prediction error of feedforward two-layer models. (TIF) [file pcbi.1006757.s012.tif]

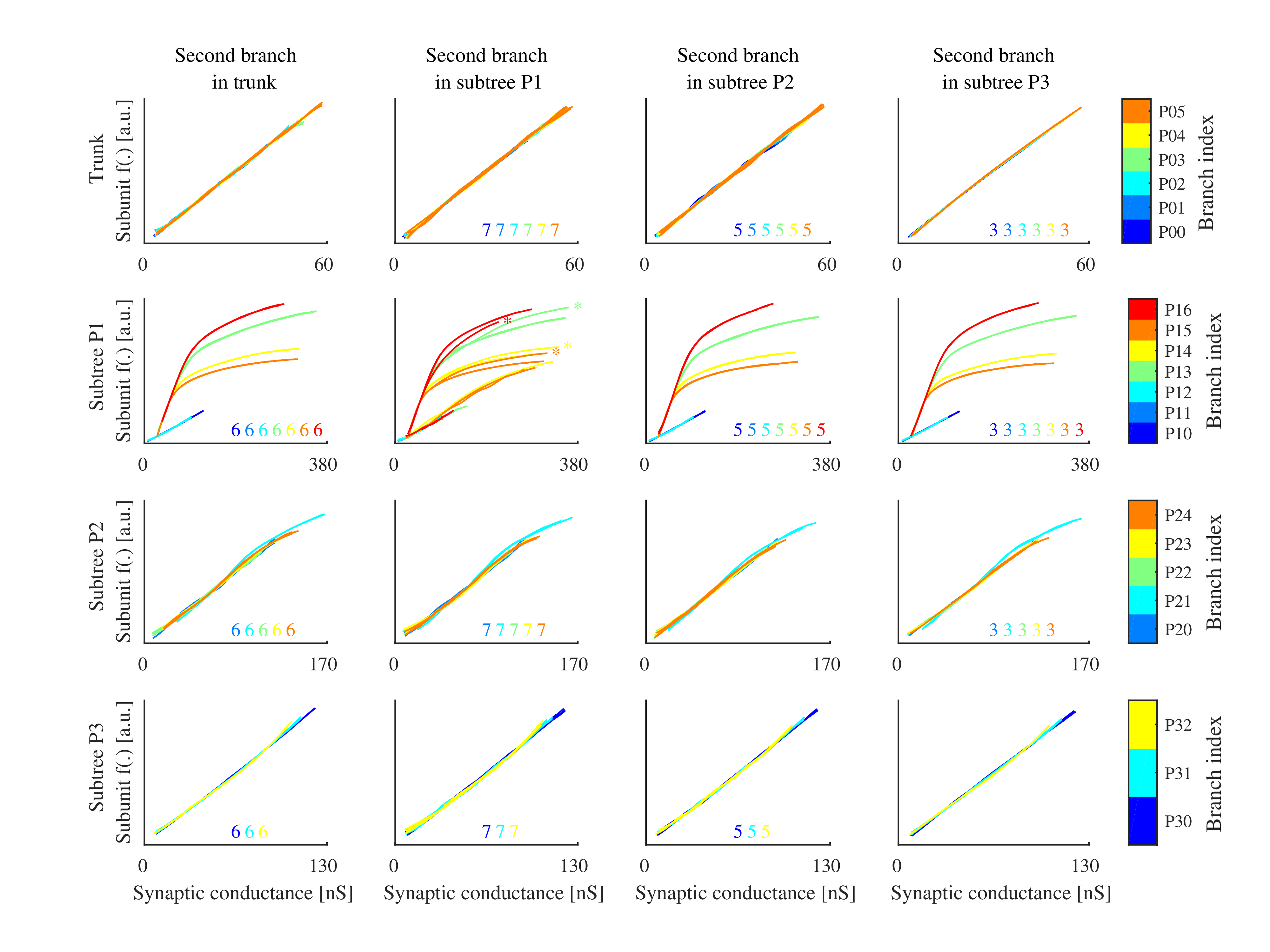

Supplement: S13 Fig — Each panel row shows the subunit functions of all dendritic branches located in the subtrees P1, P2, P3 or the trunk, respectively. Dendritic branches are indicated by colors. Color codes shown in the right most panels apply to all panels of the respective panel row. Each panel of a panel column shows the subunit functions for all partner stimulation sites (to generate iso-response curves) located in the subtrees P1, P2, P3 or the trunk, respectively. Thus, off-diagonal panels in the panel matrix correspond to dendritic branches located in different subtrees and panels on the main diagonal to branches located within the same subtree. Subunit functions with (without) linear onsets are normalized to unit slope (a slope of five) for the initial part of the function. Colored numbers indicate the number of curves shown in the same color. Subunit functions reconstructed from branch pairs located in different subtrees are independent of the location of the partner branch within the other subtree. Subunit functions reconstructed from branch pairs located in the same subtree depend on the precise location of the summation of both subunit outputs (see S6D Fig). The strongest subunit non-linearities are observed for the dendritic branches P13 to P16 when the partner branch is either P10 or P11 (and located outside of the functional compartment). The most distal dendritic branches P14 and P15 show the strongest non-linearities. Asterisks indicate subunit non-linearities reconstructed with partner branch P12. (TIF) [file pcbi.1006757.s013.tif]

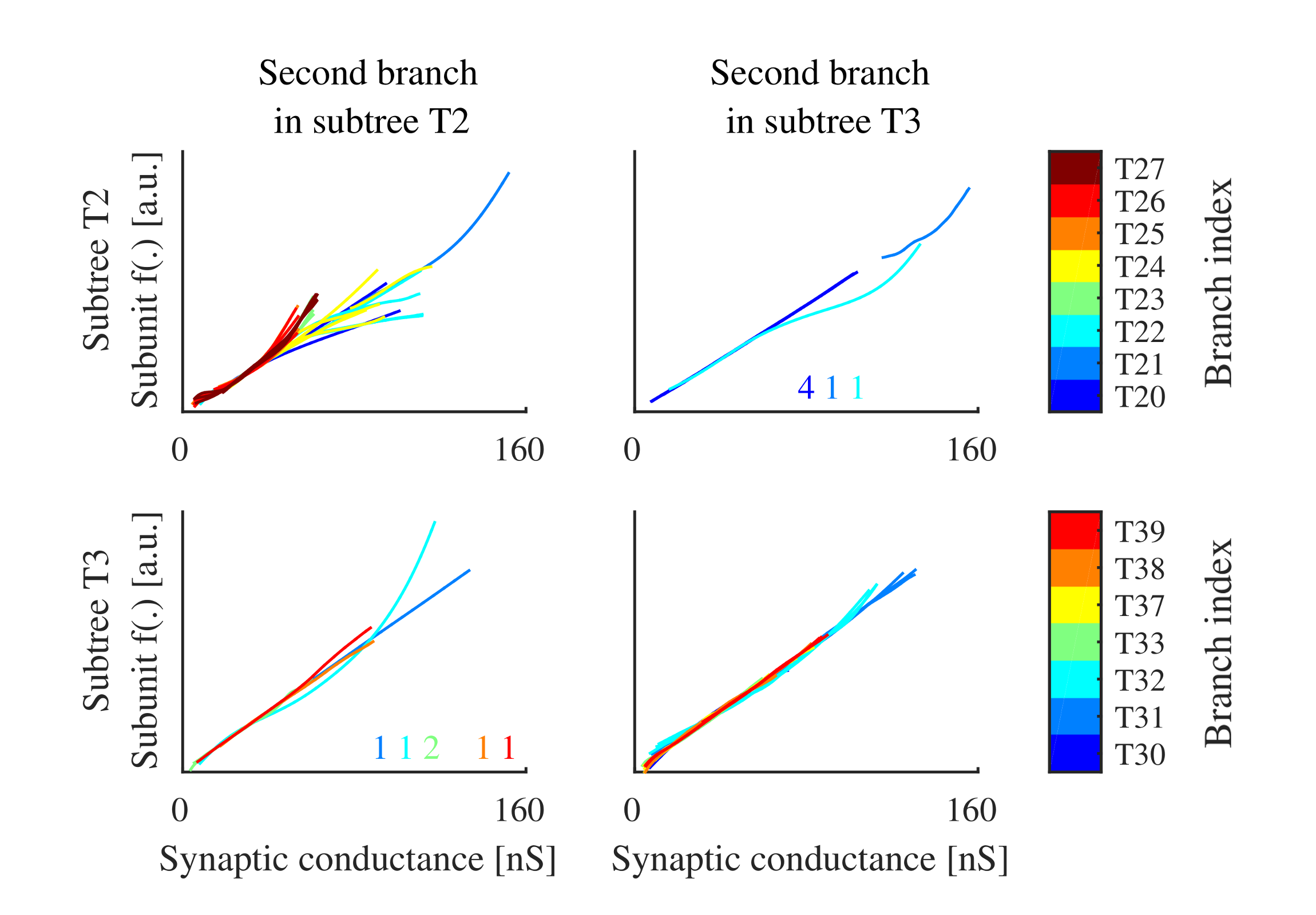

Supplement: S14 Fig — Each panel row shows the subunit functions of all dendritic branches located in the subtrees T2 or T3, respectively. Dendritic branches are indicated by colors. Color codes shown in the right most panels apply to all panels of the respective panel row. Each panel of a panel column shows the subunit functions for all partner stimulation sites (to generate iso-response curves) located in the subtrees T2 or T3, respectively. Subunit functions are normalized to unit slope for the initial part of the function. Colored numbers indicate the number of curves shown in the same color. Subunit functions reconstructed from branch pairs located in different subtrees are rare and can be considered as false positives. Subunit functions reconstructed from branch pairs located in the subtree T22-T27 are either weakly or strongly supralinear depending on the branch morphology (see S6B Fig). Subunit functions reconstructed from branch pairs located in the subtree T3 are mostly linear (parts of presumably extended non-linearities). In this case, putative linear feedback might be incorporated in a two-layer feedforward model. (TIF) [file pcbi.1006757.s014.tif]

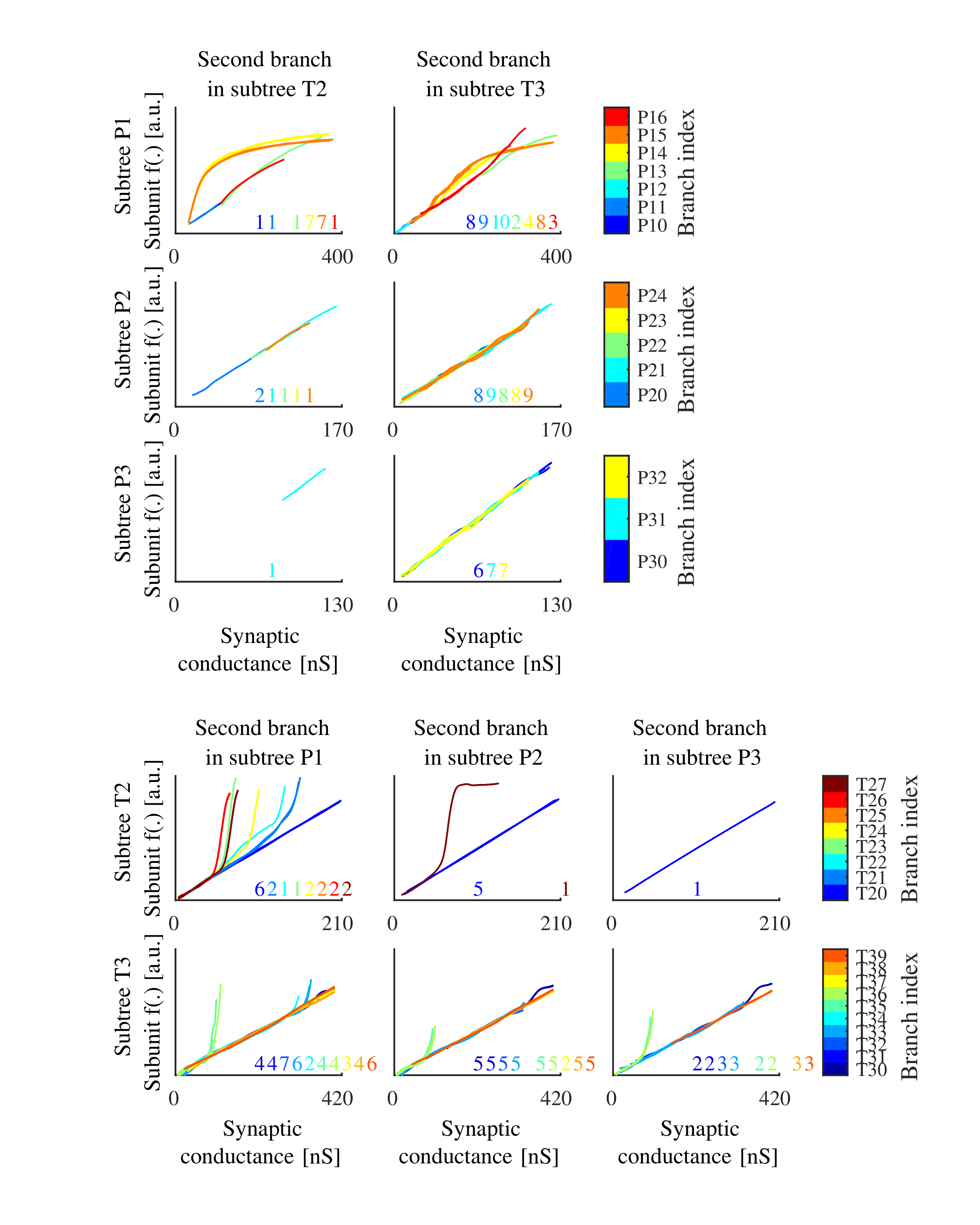

Supplement: S15 Fig — Each panel row shows the subunit functions of all selected dendritic branches located in the subtrees P1, P2, P3, T2 or T3, respectively. Dendritic branches are indicated by colors. Color codes shown in the right most panels apply to all panels of the respective panel row. Each panel of a panel column shows the subunit functions for all partner stimulation sites (to generate iso-response curves) located in the subtrees P1, P2, P3, T2 or T3, respectively. Subunit functions with (without) linear onsets are normalized to unit slope (a slope of five) for the initial part of the function. Colored numbers indicate the number of curves shown in the same color. For all panels, the more distal the dendritic branches the earlier (smaller conductance values) and stronger is the dendritic non-linearity. (TIF) [file pcbi.1006757.s015.tif]
