# Supplementary material for: Tuft dendrites of pyramidal neurons operate as feedback-modulated functional subunits
Source: PLoS Comput Biol. 2019 Mar 6;15(3):e1006757. doi: 10.1371/journal.pcbi.1006757 (PMC6402658; doi:10.1371/journal.pcbi.1006757)
Supplement: S1 Methods — Estimation of subunit non-linearities for input points that do not form a stairway. (PDF) [file pcbi.1006757.s016.pdf]

S1 Methods. Numerical estimation of subunit non-linearities. We denote the sum of the outputs of the subunit non-linearities as

$$m = f_1(s_1) + f_2(s_2). \tag{1}$$

Additionally to the response r, m is also constant on an iso-response curve. Let m_H and m_L denote the value of m on the two iso-response curves H and L, respectively. Because the iso-response method identifies f_1 and f_2 up to a linear transformation, we are free to choose $m_H - m_L = 2$. This sets the sample variance of m to one if the number of samples on both iso-response curves H and L is the same. Without loss of generality we set $f_1(0) = 0$ and $f_2(0) = 0$.

Next we use a basis-function expansion of f_1 and f_2 to approximate the subunit functions with sigmoidal basis-functions ϕ_i and ψ_i,

$$\begin{aligned} f_1(s_1) &= \sum_{m=0}^{M-1} \alpha_m \phi_m(s_1) \\ f_2(s_2) &= \sum_{n=0}^{N-1} \beta_n \psi_n(s_2). \end{aligned} \tag{2}$$

Now we can insert these expansions into Eq 1 together with $m_H - m_L = 2$ and obtain for arbitrary s_1 and s'_1

$$\sum_{m=0}^{M-1} \alpha_m(\phi_m(s_1) - \phi_m(s'_1)) \; + \\ \sum_{n=0}^{N-1} \beta_n(\psi_n(L(s_1)) - \psi_n(H(s'_1))) = 2. \tag{3}$$

Collecting the equations for many different combinations of s_1 and s'_1 we obtain a system of linear equations for the basis function coefficients α_m and β_n. This system can be solved by minimizing the mean squared error with any standard linear algebra library. Dividing two versions of Eq 3 with interchanged s_1 and s'_1 and letting $s'_1 \to s_1$ results again in Eq 3 in the main text.
